# Nuclear ATP-citrate lyase regulates chromatin-dependent activation and maintenance of the myofibroblast gene program

**DOI:** 10.1038/s44161-024-00502-3

**Published:** 2024-07-05

**Authors:** Michael P. Lazaropoulos, Andrew A. Gibb, Douglas J. Chapski, Abheya A. Nair, Allison N. Reiter, Rajika Roy, Deborah M. Eaton, Kenneth C. Bedi, Kenneth B. Margulies, Kathryn E. Wellen, Conchi Estarás, Thomas M. Vondriska, John W. Elrod

**Affiliations:** 1 Aging + Cardiovascular Discovery Center, Lewis Katz School of Medicine at Temple University, Philadelphia, PA USA; 2 Department of Anesthesiology & Perioperative Medicine, David Geffen School of Medicine at UCLA, Los Angeles, CA USA; 3 Cardiovascular Institute and Cardiovascular Medicine Division, Department of Medicine, Perelman School of Medicine at the University of Pennsylvania, Philadelphia, PA USA; 4 Department of Cancer Biology, University of Pennsylvania Perelman School of Medicine, Philadelphia, PA USA; 5 Departments Medicine/Cardiology and Physiology, and Molecular Biology Institute, David Geffen School of Medicine at UCLA, Los Angeles, CA USA

**Keywords:** Cell signalling, Chromatin remodelling, Heart failure, Metabolomics

## Abstract

Differentiation of cardiac fibroblasts to myofibroblasts is necessary for matrix remodeling and fibrosis in heart failure. We previously reported that mitochondrial calcium signaling drives α-ketoglutarate-dependent histone demethylation, promoting myofibroblast formation. Here we investigate the role of ATP-citrate lyase (ACLY), a key enzyme for acetyl-CoA biosynthesis, in histone acetylation regulating myofibroblast fate and persistence in cardiac fibrosis. We show that inactivation of ACLY prevents myofibroblast differentiation and reverses myofibroblasts towards quiescence. Genetic deletion of *Acly* in post-activated myofibroblasts prevents fibrosis and preserves cardiac function in pressure-overload heart failure. TGFβ stimulation enhances ACLY nuclear localization and ACLY–SMAD2/3 interaction, and increases H3K27ac at fibrotic gene loci. Pharmacological inhibition of ACLY or forced nuclear expression of a dominant-negative ACLY mutant prevents myofibroblast formation and H3K27ac. Our data indicate that nuclear ACLY activity is necessary for myofibroblast differentiation and persistence by maintaining histone acetylation at TGFβ-induced myofibroblast genes. These findings provide targets to prevent and reverse pathological fibrosis.

## Abstract (web summary)

Elrod and colleagues reveal the role of ATP-citrate lyase in myofibroblast differentiation and cardiac fibrosis.

## Main

Excessive cardiac fibrosis is the pathological consequence of an initially reparative process following cardiac injury^1^. Resident cardiac fibroblasts (CFs) respond to stress, such as pressure overload from hypertension and ischemic injury from myocardial infarction, resulting in fibroblast activation and differentiation^2,3^, which is a major contributor to pathological cardiac remodeling and heart failure (HF)^4^. Initial fibroblast activation results in migration to the site of injury, a proliferative burst, differentiation into myofibroblasts and increased extracellular matrix (ECM) production and secretion^5,6^. Fibroblasts are activated by a variety of secreted, pro-fibrotic factors such as transforming growth factor-β (TGFβ), angiotensin II and endothelin 1, in addition to environmental cues such as mechanical tension and hypoxia^7–10^. Sustained stress, as occurs in cardiovascular disease, causes activated fibroblasts to maintain the myofibroblast phenotype, characterized by unremitting ECM secretion and the expression of alpha-smooth muscle actin (αSMA), a contractile stress protein^11^. While myofibroblasts are essential for tissue repair, their prolonged presence in the heart contributes to ECM expansion, resulting in myocardial stiffening and loss of ventricular compliance^12,13^. Recent evidence suggested that reversal of the myofibroblast phenotype in formerly activated CFs could occur following withdrawal of stress stimuli^14^. Work from our laboratory directly showed myofibroblast reversal towards a less fibrotic phenotype by inhibiting glutaminolysis, a key metabolic pathway mediating myofibroblast formation and persistence^15,16^. These studies are proof of concept for therapeutic strategies targeting the signaling pathways contributing to myofibroblast fate and persistence to reduce cardiac fibrosis in HF^17^.

Changes in metabolism and metabolite signaling are appreciated as central mediators of cell fate transitions, such as myofibroblast differentiation^18–20^. Metabolic remodeling is necessary not only to support the biosynthetic and bioenergetic demands necessary for cellular differentiation, but also supplies the cofactors necessary for the activity of epigenetic modifiers controlling gene expression and cell lineage^21–23^. Recently, our laboratory identified the requirement of glutaminolysis and increases in alpha-ketoglutarate bioavailability for histone-lysine-demethylase-dependent activation of the myofibroblast gene program^16,18^. While histone demethylation represents one means to activate changes in the fibroblast gene program, several other epigenetic mechanisms work in concert to control gene expression. Indeed, our pairwise omics datasets examining chromatin remodeling by assay for transposase-accessible chromatin sequencing and transcription by RNA sequencing identified that while chromatin accessibility was necessary for the overall changes in gene expression that occur with myofibroblast formation, it did not distinguish between upregulated or downregulated genes^16^. This discrepancy highlights that while histone-demethylation-dependent changes in chromatin accessibility are necessary for myofibroblast formation and maintenance, other mechanisms are controlling and reinforcing loci-specific changes in gene expression (that is, the upregulation or downregulation of transcription). Acetylation is dependent on the cofactor acetyl-CoA, which is used by histone acetyltransferases (HATs) to affix electronegative acetyl groups on histone tail lysines to regulate chromatin-dependent gene transcription^24^. Previous work has shown how acetyl-CoA supply for histone acetylation and chromatin remodeling influences cellular fate decisions^19,21,25^. However, whether the enzymatic activity of acetyl-CoA-synthesizing enzymes, such as ATP-citrate lyase (ACLY), is necessary for stress-responsive myofibroblast differentiation at the level of the epigenome remains unknown and represents a plausible mechanism controlling loci-specific gene regulation. Here, we tested the hypothesis that ACLY regulates histone acetylation and chromatin remodeling to initiate and maintain the myofibroblast gene program and fibrotic phenotype.

## Results

### ACLY is necessary for myofibroblast differentiation

**Fig. 1:**
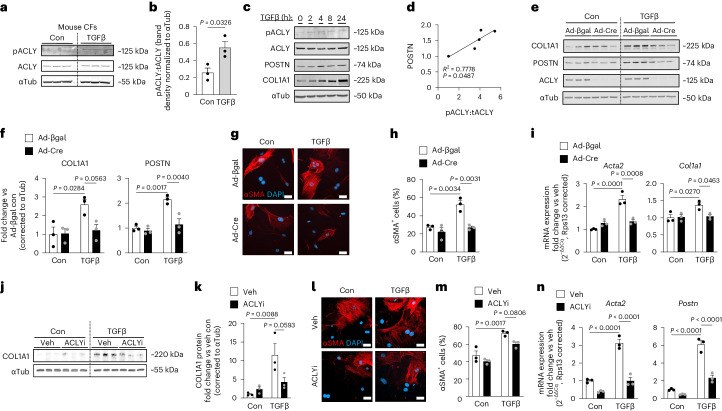
Increased ACLY activity is necessary for myofibroblast differentiation. **a**, Western blot of mouse CFs with and without TGFβ treatment for 48 h. Immunoblots of pSer455–ACLY (pACLY), ACLY and αTubulin (αTub), loading control (load con). **b**, pACLY to total ACLY (tACLY) protein expression, band intensity (arbitrary units) ratio normalized to αTub from **a**. Unpaired two-sided *t-*test. **c**, Western blot of CFs treated with and without TGFβ for 0–24 h. Immunoblots for pACLY, ACLY, POSTN, COL1A1 and αTub. **d**, Correlation of POSTN and the pACLY:tACLY ratio (*n* = 3 biological replicates per timepoint). **e**, Western blot of COL1A1, POSTN and αTub (load con). CFs were transduced with adenovirus expressing Cre or βgal and treated with TGFβ or vehicle (Con). **f**, COL1A1 and POSTN protein expression normalized to αTub. **g**, Myofibroblasts identified by αSMA immunostaining (red), co-stained with DAPI (blue). Scale bars, 50 μm. **h**, Percentage of αSMA^+^ cells. **i**, qPCR mRNA expression of *Acta2* and *Col1a1*. **j**, CFs treated with ACLYi (BMS-303141, 4 μM) or veh, with or without 10 ng ml^−1^ TGFβ, for 48 h; immunoblots of COL1A1 and αTub (load con). **k**, COL1A1 protein expression normalized to αTub. **l**, Myofibroblasts identified by αSMA immunostaining (red), co-stained with DAPI (blue). Scale bars, 50 μm. **m**, Percentage of αSMA^+^ cells. **n**, qPCR mRNA expression of *Acta2* and *Postn*. *n* = 3 biological replicates. All data are depicted as mean ± s.e.m. Two-way ANOVA with Tukey honestly significant difference (HSD) for multiple comparisons. Veh, vehicle. Full-length western blots are available as source data. Source data

To determine whether ACLY expression or activation is enhanced during myofibroblast differentiation, mouse primary CFs were treated with and without the pro-fibrotic agonist TGFβ for 48 h and examined for changes in ACLY protein expression and phosphorylation. CFs showed a significant increase in ACLY phosphorylation at Ser455 following TGFβ stimulation (Fig. 1a,b). Phosphorylation at Ser455 is reported to increase the catalytic activity of ACLY, the conversion of citrate to acetyl-CoA and oxaloacetate^26–28^. CFs were stimulated with TGFβ for 2–24 h and ACLY phosphorylation correlated with time-dependent changes in periostin (POSTN) protein expression, which is an ECM protein that is upregulated early in fibroblast activation and a marker of myofibroblast fate (Fig. 1c,d). To determine whether ACLY is necessary for myofibroblast differentiation, mouse embryonic fibroblasts (MEFs) were isolated from mice containing *loxP* sites flanking exon 9 of *Acly*^29^, permitting conditional deletion following transduction with adenovirus-encoding Cre recombinase (Ad-Cre). MEFs treated with Ad-Cre resulted in a near complete loss of ACLY protein (Fig. 1e). Loss of ACLY prevented TGFβ-mediated myofibroblast formation as indicated by a decrease in collagen type I alpha 1 (COL1A1) and POSTN expression (Fig. 1e,f) and the population of αSMA^+^ myofibroblasts (Fig. 1g,h). At the level of gene transcription, loss of ACLY prevented the upregulation of alpha actin 2 (*Acta2*) and *Col1a1* (Fig. 1i), suggesting a direct role for ACLY in activating the fibrotic gene program underlying myofibroblast formation. To validate these results, we treated primary mouse CFs with BMS-303141 (4 μM), a highly specific pharmacological inhibitor of ACLY^30^ (ACLYi) at the time of TGFβ stimulation. Pharmacological inhibition of ACLY was sufficient to prevent myofibroblast differentiation indicated by reduced COL1A1 expression (Fig. 1j,k), a decreased percentage of αSMA^+^ fibroblasts (Fig. 1l,m) and reduced expression of fibrotic genes (Fig. 1n). These data show that ACLY activity is necessary for TGFβ-mediated myofibroblast differentiation and suggest a primary role at the level of gene activation.

### ACLY inhibition reverts the myofibroblast phenotype

**Fig. 2:**
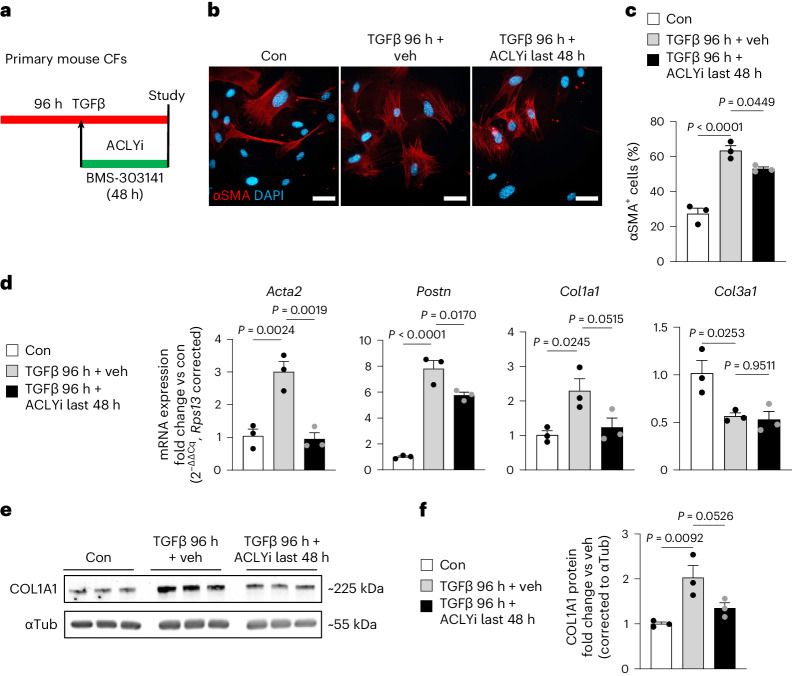
ACLY inhibition reverts the myofibroblast phenotype, even with continuous TGFβ stimulation. **a**, Timeline of reversion treatments with 4 μM BMS-303141 (ACLYi) in vitro on adult mouse CFs. Con, control. **b**, Myofibroblasts identified by αSMA by immunostaining (red), co-stained with DAPI (blue). Scale bars, 50 μm. **c**, Percentage of αSMA^+^ cells. **d**, qPCR mRNA expression of *Acta2*, *Postn*, *Col1a1* and *Col3a1*. **e**, Immunoblots of COL1A1 and αTubulin (load con). **f**, COL1A1 protein expression normalized to αTubulin. *n* = 3 biological replicates. All data are depicted as mean ± s.e.m. One-way ANOVA with Dunnett’s HSD. Full-length western blots are available as source data. Source data

**Extended Data Fig. 1:**
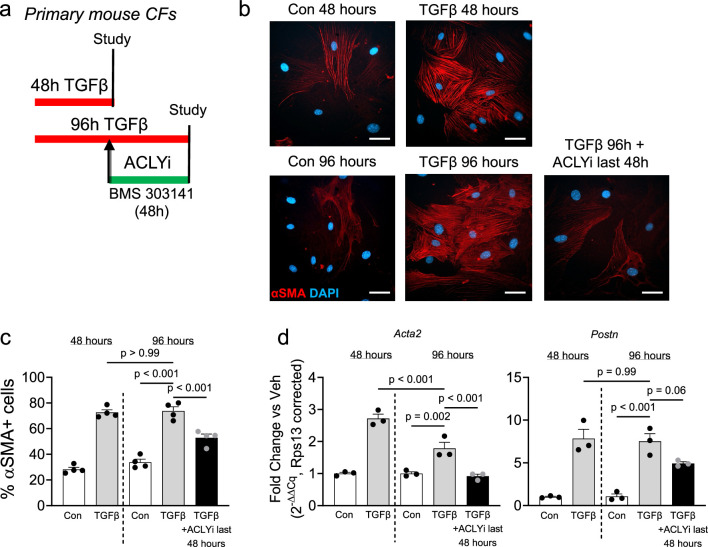
ACLY inhibition after the onset of differentiation reverses myofibroblast phenotype. **(a)** Timeline of reversion treatments with isolation of CFs at the 48 h timepoint before adding 4μM BMS-303141 (ACLYi) *in vitro* on adult mouse CFs. Control (Con). **(b)** Myofibroblasts identified by αSMA by immunostaining (red), co-stained with DAPI (blue). Scale bars = 50 mm. **(c)** Percent αSMA+ cells. n = 4 biological replicates. **(d)** qPCR mRNA expression of Alpha Actin 2 (Acta2), periostin (Postn), n = 3 biological replicates. All data depicted as mean ± SEM. One-way ANOVA with Dunnett’s HSD against TGFβ 96 h.

Clinically successful resolution of pathological fibrosis will require deactivating the myofibroblast’s pro-fibrotic phenotype to a less pathogenic state. Therefore, we tested whether CFs already differentiated into myofibroblasts, and under continuous TGFβ stimulation, could be dedifferentiated to a less fibrotic phenotype with ACLY intervention. Primary CFs were stimulated with and without TGFβ for 96 h, with ACLYi introduced at the 48 h mark, a timepoint at which myofibroblast differentiation and formation is significantly increased and maximal (Fig. 2a). Addition of the ACLYi at the 48 h mark decreased the percentage of αSMA^+^ myofibroblasts (Fig. 2b,c). ACLYi also reversed TGFβ-mediated upregulation of *Acta2*, *Postn* and *Col1a1*, but did not reverse the observed decrease in collagen type III alpha 1 (*Col3a1*; Fig. 2d), suggesting that ACLY may infer a level of specificity in gene regulation. Lastly, ACLYi also decreased COL1A1 protein abundance in CFs (Fig. 2e,f). To determine whether ACLY inhibition at the 48 h mark was truly reverting myofibroblast activation or preventing further myofibroblast conversions from that timepoint onward, we repeated these experiments with additional samples of control and TGFβ-stimulated CFs collected at the 48 h timepoint to compare the extent of myofibroblast differentiation at 48 h versus 96 h (Extended Data Fig. 1a). This approach revealed that the proportion of αSMA-positive fibroblasts was equivalent between 48 h and 96 h of TGFβ treatment (Extended Data Fig. 1b,c). mRNA expression of *Postn* was also increased to a similar level at 48 h and 96 h, and *Acta2* mRNA expression was slightly higher at 48 h than at 96 h (Extended Data Fig. 1d). Collectively, these data suggest a necessary role for ACLY in mediating not only myofibroblast formation but also the persistence of the myofibroblast phenotype, providing a therapeutic target to reverse the pathological phenotype of these cells in the context of tissue fibrosis.

### Genetic deletion of *Acly* in CFs decreases cardiac fibrosis

**Fig. 3:**
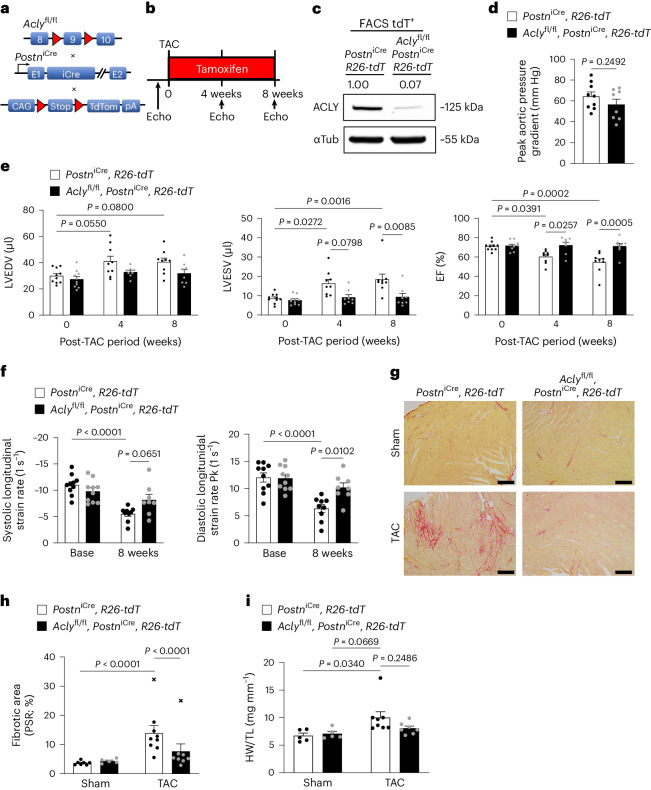
*Acly* deletion in cardiac activated myofibroblasts preserves cardiac function and reduces interstitial fibrosis. **a**, Tamoxifen-inducible, Cre-recombinase-mediated gene knockout of *Acly* and expression of R26-tdT in activated fibroblasts. **b**, Mice subjected to TAC at 0 week and started on tamoxifen chow to induce Cre-recombinase expression in activated CFs. Echocardiograms (Echo) performed every 4 weeks are indicated by arrows. Take-downs were performed at 8 weeks for interstitial fibrosis and gravimetrics. **c**, Western blot of ACLY knockout in *Acly*^fl/fl^, *Postn*^iCre^, *R26-tdT* CFs. **d**, Peak aortic pressure gradients from *Postn*^iCre^, *R26-tdT* mice and *Acly*^fl/fl^, *Postn*^iCre^, *R26-tdT* mice that underwent TAC. Unpaired two-sided *t*-test. *Postn*^iCre^, *R26-tdT* + TAC = 9 mice; *Acly*^fl/fl^, *Postn*^iCre^, *R26-tdT* + TAC = 8 mice. **e**, Echocardiographic data of mice followed 8 weeks after TAC. Long-axis, B-mode images. EF, ejection fraction; LVESV, LV end-systolic volume; LVEDV, LV end-diastolic volume. Two-way ANOVA with Tukey HSD. Comparisons between *Postn*^iCre^, *R26-tdT* mice and *Acly*^fl/fl^, *Postn*^iCre^*, R26-tdT* mice at the same timepoint. Comparisons between different timepoints of the same genotype. *Postn*^iCre^, *R26-tdT* + TAC = 10 mice at 0 week and 9 mice at 4 weeks and 8 weeks; *Acly*^fl/fl^, *Postn*^iCre^, *R26-tdT* + TAC = 10 mice at 0 week and 8 mice at 4 weeks and 8 weeks. **f**, B-mode speckle tracking calculations of LV strain rates at baseline (Base) and 8 weeks after TAC. Two-way ANOVA with Tukey HSD. *Postn*^iCre^, *R26-tdT* + TAC = 10 mice at baseline and 9 mice at 8 weeks; *Acly*^fl/fl^, *Postn*^iCre^, *R26-tdT* + TAC = 10 mice at 0 week and 8 mice at 8 weeks. **g**, Picrosirius red staining of matrix protein in 10 μm LV sections. Scale bars, 200 μm. **h**, Percentage of the picrosirius red (PSR) stain over the total area. Outliers were identified by Grubbs test with an alpha of 0.05 and indicated with ‘×’ in the graph. Two-way ANOVA with Tukey HSD. **i**, Gravimetric data from mice at 8 weeks after TAC. HW, heart weight; TL, tibia length. Two-way ANOVA with Tukey HSD. For **h** and **i**, *Postn*^iCre^, *R26-tdT* + sham = 7 mice; *Acly*^fl/fl^, *Postn*^iCre^, *R26-tdT* + sham = 5 mice; *Postn*^iCre^, *R26-tdT* + TAC = 8 mice; and *Acly*^fl/fl^, *Postn*^iCre^, *R26-tdT* + TAC = 9 mice. All data are depicted as mean ± s.e.m. Full-length western blots are available as source data. Source data

**Extended Data Fig. 2:**
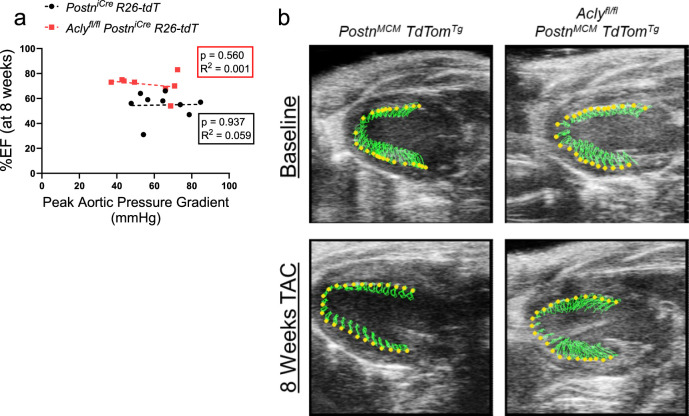
Aortic Pressure Gradients and B-mode speckle tracking on transgenic mice. **(a)** Linear regression of peak aortic pressure gradients and %EF at 8 weeks from *Acly*^fl/fl^*, Postn*^iCre^*, R26-tdT* and *Postn*^iCre^*, R26-tdT* mice undergoing TAC. **(b)** Echocardiography images trace the path (green lines) that individual speckles (yellow dots) were tracked during cycles of systole and diastole in *Acly*^fl/fl^*, Postn*^iCre^*, R26-tdT* and *Postn*^iCre^*, R26-tdT* mice.

To determine whether loss of ACLY was sufficient to prevent the myofibroblast phenotype and mitigate cardiac fibrosis and functional decline in response to cardiac injury, we genetically deleted *Acly* exclusively in activated, post-differentiated myofibroblasts in a mouse model of pressure-overload-induced HF. *Acly*^fl/fl^ mice were crossed with the tamoxifen-inducible *Postn*^iCre^ mouse model, which restricts Cre recombinase expression to activated and differentiated CFs. These mice were then crossed with the Rosa26-TdTomato reporter line (*R26-tdT*) to fluorescently label and permit isolation of myofibroblasts throughout the course of study (Fig. 3a). Baseline left ventricular (LV) function was examined by echocardiography followed by randomization to sham or transverse aortic constriction (TAC) surgery of control (*Postn*^iCre^, *R26-tdT*) and experimental (*Acly*^fl/fl^, *Postn*^iCre^, *R26-tdT*) mice. Tamoxifen citrate chow was administered to all groups for the duration of the study, as fibroblast activation is occurring continuously throughout disease progression, and cardiac function was monitored by echocardiography 4 weeks and 8 weeks after TAC (Fig. 3b). Fluorescence-activated cell sorting (FACS)-isolated TdTomato^+^ myofibroblasts were probed for ACLY expression by western blot for efficient Cre-mediated *Acly* deletion (Fig. 3c). Both genotypes showed equivalent peak aortic pressure gradients across the constriction (Fig. 3d and Extended Data Fig. 2a) 1 week after TAC, indicating equivalent aortic ligation. Loss of ACLY in activated myofibroblasts reduced adverse left ventricular remodeling (LV end-diastolic and end-systolic volumes) and preserved the ejection fraction (EF%) compared with control mice, both at the 4 week and 8 week timepoints (Fig. 3e). The B-mode speckle tracking 8 weeks after TAC revealed that longitudinal LV strain rates in *Acly*^fl/fl^, *Postn*^iCre^*,* *R26-tdT* mice were higher than those in *Postn*^iCre^*, R26-tdT* control mice during both systole and diastole (Fig. 3f and Extended Data Fig. 2b), suggesting greater myocardial contractility and relaxation, respectively. Improved longitudinal strain rates are noteworthy, as reduced longitudinal diastolic strain is an indicator of reduced LV compliance, which correlates with cardiac fibrosis^31^. To assess whether loss of ACLY in activated myofibroblasts prevented ECM deposition, heart cross sections were stained with picrosirius red and the fibrotic area was quantified. Loss of ACLY in myofibroblasts decreased LV interstitial collagen deposition (percentage of the fibrotic area) compared with the control (Fig. 3g,h). Despite preserved cardiac function and decreased interstitial fibrosis, there was no significant decrease in cardiac hypertrophy (heart weight to tibia length ratio) with deletion of *Acly* in activated CFs (Fig. 3i). These data suggest that loss of ACLY activity, specifically in activated, pro-fibrotic CFs, is sufficient to decrease interstitial fibrosis and preserve cardiac function during HF progression.

### ACLY translocates to the nucleus with fibrotic stimulation

**Fig. 4:**
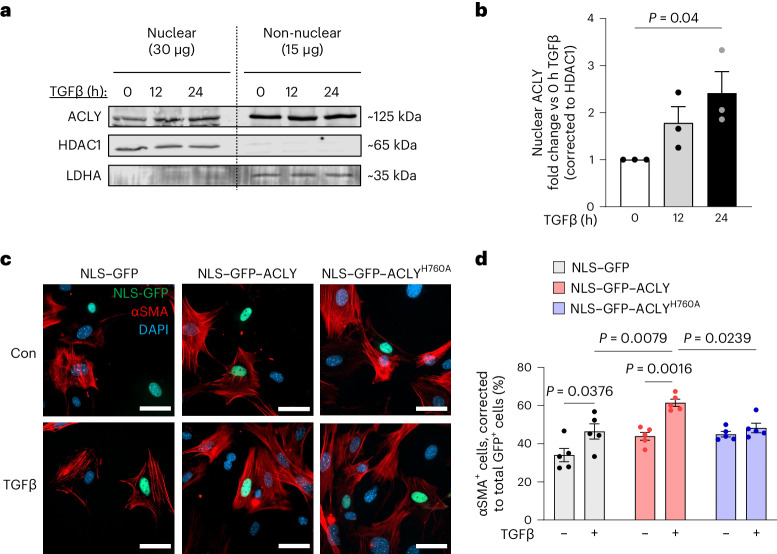
Nuclear ACLY activity is essential to TGFβ-dependent myofibroblast activation. **a**, Nuclear fractionation of immortalized CFs stimulated with TGFβ for 12 h and 24 h. Immunoblot for ACLY, histone deacetylase 1 (HDAC1) as the nuclear loading control, and lactate dehydrogenase A (LDHA) as the non-nuclear loading control. **b**, Nuclear ACLY protein expression normalized to HDAC1. One-way ANOVA with Dunnett’s HSD with 0 h TGFβ as the control, *n* = 3 biological replicates. **c**, MEFs transfected with NLS–GFP control, NLS–GFP–ACLY and NLS–GFP–ACLY^H760A^ (all green) treated with and without TGFβ. Myofibroblasts identified by αSMA immunostaining (red), co-stained with DAPI (blue). Scale bars, 50 μm. **d**, Percentage of αSMA^+^ cells among GFP^+^ cells. All data are depicted as mean ± s.e.m. Two-way ANOVA with Tukey’s HSD. *n* = 5 biological replicates. Full-length western blots are available as source data. Source data

**Extended Data Fig. 3:**
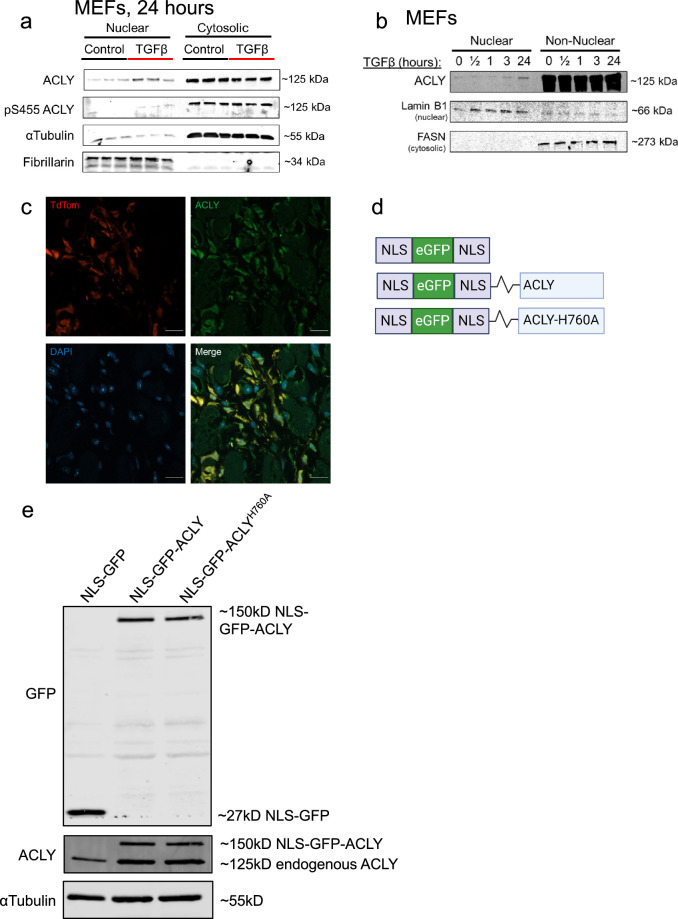
ACLY localizes to the nucleus with TGFβ stimulation. **(a)** Nuclear fractionation of MEFs stimulated with TGFβ for 24 h. Immunoprobed for total ACLY, pSer455-ACLY (pACLY), fibrillarin as the nuclear loading control and αTubulin as the cytosolic control. n = 3 biological replicates per group. **(b)** Nuclear fractionation of MEFs stimulated with TGFβ over a time course of 0.5 h to 24 h. Immunoblot for total ACLY, Lamin B1 as the nuclear loading control, and fatty acid synthase (FASN) as the non-nuclear loading control. **(c)** Representative immunofluorescence images of tissue section from *Postn*^iCre^*, R26-tdT* mouse that underwent TAC demonstrate that TdTomato+ activated fibroblasts (red) contain ACLY (green) in nuclei (DAPI, blue). Scale bar = 50 μm. **(d)** Fusion protein constructs utilized in Fig. 4c. **(e)** Western blot of MEFs transfected with plasmids containing fusion protein constructs. Panel **d** created with BioRender.com. Full length western blots in Source Data Fig. 6. Source data

Nuclear ACLY has previously been shown to regulate histone acetylation^32^ via the catalytic conversion of citrate to acetyl-CoA by ACLY, a necessary cofactor for HATs. However, whether ACLY nuclear localization and activity is necessary for myofibroblast differentiation is unknown. To examine the localization of ACLY in quiescent, activated and differentiated fibroblasts, we examined fibroblasts with and without TGFβ at varying timepoints and performed nuclear fractionation for western blotting. A low level of nuclear ACLY was observed in unstimulated CFs and was increased after 12 h and 24 h of TGFβ stimulation (Fig. 4a,b). Similar observations were made in MEFs, with nuclear ACLY accumulation occurring as early as 3 h after TGFβ treatment (Extended Data Fig. 3a,b). In addition, immunohistological analysis of pressure-overload mouse hearts revealed that ~92% of TdTomato^+^ activated CFs (Postn-dependent labeling) exhibited ACLY expression in the nucleus (Extended Data Fig. 3c). To determine whether forcing nuclear localization of ACLY was sufficient for myofibroblast differentiation, we created a plasmid encoding an ACLY–eGFP fusion construct containing a canonical nuclear localization sequence (NLS), and to test whether nuclear ACLY activity is necessary for myofibroblast formation, we designed a plasmid encoding eGFP fused to catalytically dead ACLY (H760A), again with a canonical NLS (Extended Data Fig. 3d,e). Histidine 760 on ACLY is required for its catalytic activity to convert citrate to phospho-citrate, and substitution of this residue for alanine has been shown to render ACLY inactive^33,34^. MEFs transfected with NLS–eGFP–ACLY fusion constructs were treated with TGFβ for 48 h, and differentiation was assessed by quantifying the percentage of eGFP^+^ cells that were also αSMA^+^ (Fig. 4c). Transfection of primary fibroblasts with the NLS–GFP–ACLY construct alone was not sufficient to promote differentiation, but this construct did potentiate TGFβ-mediated differentiation (Fig. 4c,d). Transfection with the catalytically dead NLS–GFP–ACLY^H760A^ construct failed to increase the population of αSMA^+^ myofibroblasts in response to TGFβ (Fig. 4c,d), suggesting that ACLY catalytic activity in the nucleus is necessary for myofibroblast differentiation. These data suggest that nuclear ACLY activity is required for myofibroblast differentiation induced by TGFβ.

### ACLY regulates H3K27ac at fibrotic gene loci

**Fig. 5:**
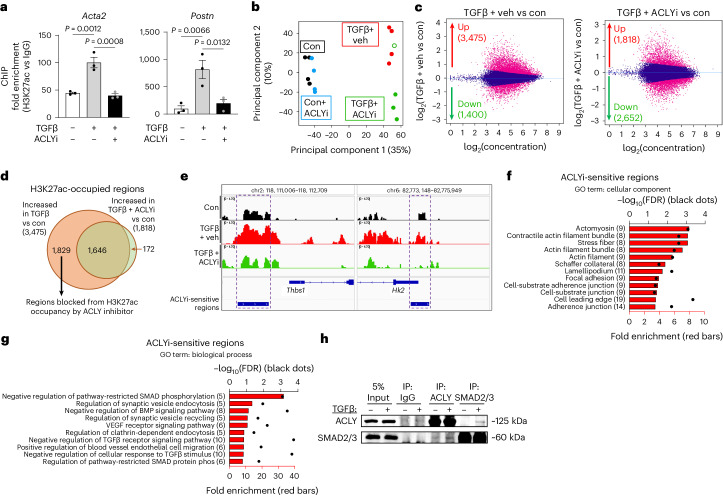
H3K27ac at fibrotic gene program loci requires ACLY activity. **a**, ChIP–qPCR with H3K27ac antibody (ab4729) regulatory regions of *Acta2* and *Postn*. Normalized to Ct values of rabbit IgG. One-way ANOVA with Dunnett’s HSD with TGFβ DMSO as positive control, *n* = 3 biological replicates. Data are depicted as mean ± s.e.m. **b**, Principal component analysis showing two principal components (PC1 and PC2) for H3K27ac-occupied regions for control (con; *n* = 4), con + ACLYi (*n* = 4), TGFβ + veh (*n* = 4) and TGFβ + ACLYi (*n* = 3). Cohorts are color coded, and each sample is represented by an individual point. The open circle represents a sample removed from downstream analysis. **c**, CUT&RUN-seq H3K27ac MA plots comparing TGFβ + veh versus con and TGFβ + ACLY versus con. Pink dots indicate regions with significant change in occupancy (FDR < 0.05). **d**, Venn diagram of H3K27ac-occupied regions increased in TGFβ versus con (orange) and regions increased in TGFβ + ACLYi versus con (green). The 1,829 regions that were increased only between TGFβ and con were examined for further study. **e**, Genome browser viewing tracks of ACLYi-sensitive regions outlined by dashed lines. Representative genes thrombospondin 1 (*Thbs1*) and hexokinase 2 (*Hk2*). **f**, DAVID gene ontology enrichment analysis of the 253 genes corresponding to the 1,829 ACLYi-sensitive regions, representing cellular components. The bottom *x*-axis represents the fold enrichment of genes in red bars of interest over a mouse genome background. The numbers in parentheses are the number of genes aligned to the GO term. The top *x*-axis represents −log_10_(FDR) by dot plot of genes of interest over a mouse genome background. **g**, DAVID gene ontology enrichment analysis of the 253 genes corresponding to the 1,826 ACLYi-sensitive regions representing biological processes. BMP, bone morphogenetic protein; VEGF, vascular endothelial growth factor. **h**, Co-immunoprecipitation of immortalized CF lysate treated with and without TGFβ for 12 h. Full-length western blots are available as source data. Source data

**Extended Data Fig. 4:**
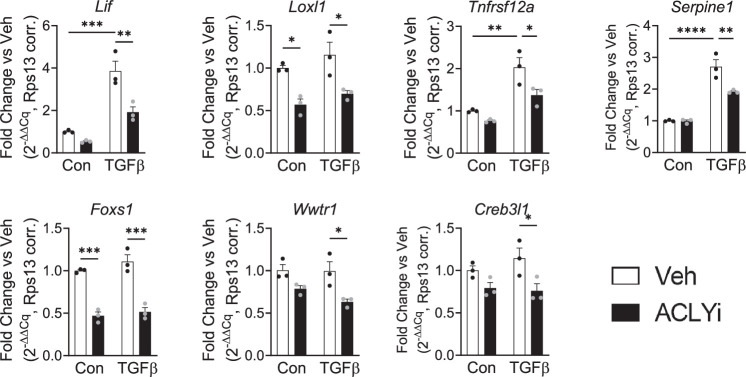
mRNA expression of genes whose TGFβ-dependent H3K27ac-occupancy was blocked by ACLYi. Mouse CFs co-treated with or without 10 ng/mL TGFβ and 4μM ACLY inhibitor (BMS 303141; ACLYi) for 48 h. Control (Con). Gene expression analysis using qPCR. Leukemia Inhibitory Factor (*Lif*), Lysyl Oxidase Like 1 (*Loxl1*), TNF Receptor Superfamily Member 12 A (*Tnfrs12a*), Serpin Family E Member 1 (*Serpine1*), Forkhead Box S1 (*Foxs1*), WW Domain Containing Transcription Regulator 1 (*Wwtr1*), cAMP Responsive Element Binding Protein 3 Like 1 (*Creb3l1*). n = 3 biological replicates. All bar graphs represented as mean with SEM for error bars. Two-way ANOVA with Tukey HSD for multiple comparisons.

**Extended Data Fig. 5:**
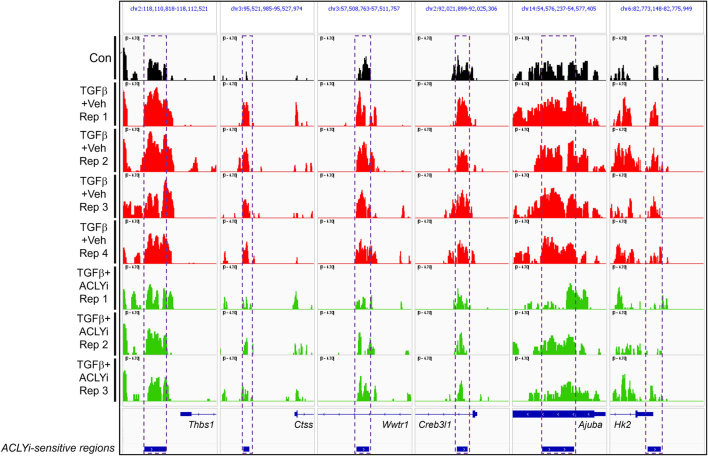
TGFβ-induced H3K27ac-enriched CUT&RUN DNA sensitive to ACLY inhibition. IGV tracks view with an y-axis of 0 to log_10_(4.70) with x-axis genomic coordinates provided above tracks. Control (Con), TGFβ+Vehicle (TGFβ+Veh). From left to right, Thrombospondin 1 (*Thbs1*)^65^, Cathespin S (*Ctss*)^66^, WW domain containing transcription regulator 1(*Wwtr1*)^42^, cAMP responsive element binding protein 3 like 1 (*Creb3l1*)^41^, LIM Domain-Containing Protein Ajuba (*Ajuba*)^67^, Hexokinase (*Hk2*)^68^. Dashed lines indicate which peaks/region are sensitive to ACLY inhibition.

**Extended Data Fig. 6:**
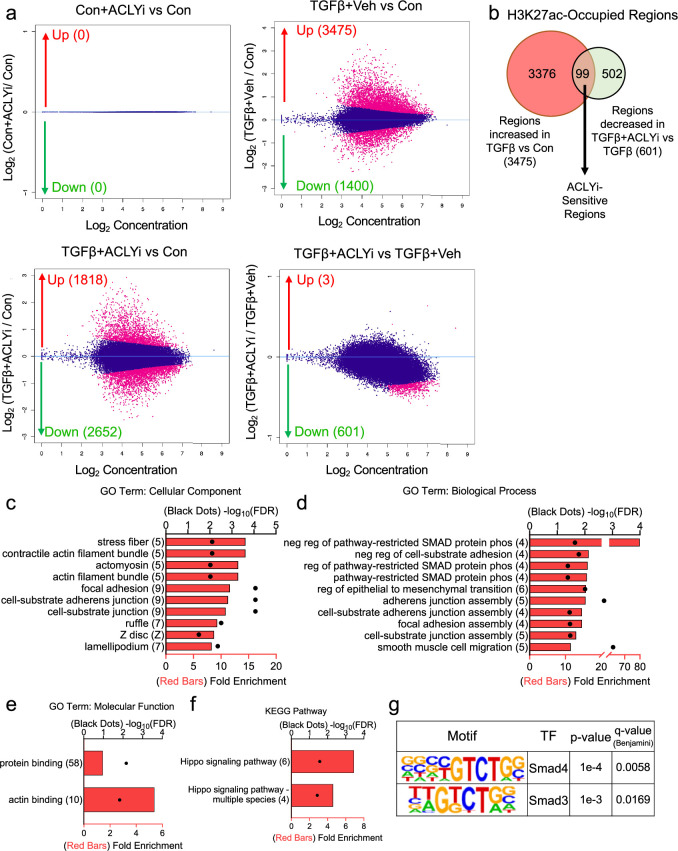
H3K27ac occupancy dependent on ACLY and TGFβ. **(a)** CUT&RUN-Seq H3K27ac MA plots of all comparisons made between the four groups in Fig. 5b. Control (Con), Vehicle (Veh). Upper left compares ACLYi+Con vs Con, upper right compares TGFβ+Veh vs Con (reproduced from Fig. 5c), lower left compares TGFβ+ACLYi vs Con (reproduced from Fig. 5c), and lower right compares TGFβ+ACLYi vs TGFβ+Veh. Pink dots indicate regions with significant change in occupancy (FDR < 0.05). **(b)** Venn Diagram of H3K27ac occupied regions increased in TGFβ vs Con (red) and regions decreased in TGFβ+ACLYi vs TGFβ+Veh (green). The 99 regions which overlapped in these two comparisons were examined for further study. DAVID Gene Ontology enrichment analysis of the 89 genes corresponding to the 99 overlapping regions from (b), with **(c)** representing Cellular Components and **(d)** representing Biological Processes. Bottom x-axis represents the fold enrichment of genes in red bars of interest over mouse genome background. Parentheses give the number of genes aligned to the GO term. Top x-axis represents –log_10_(FDR) by dot plot of genes of interest over mouse genome background. DAVID gene ontology enrichment analysis of the 89 genes corresponding to the 99 overlapping regions from (b), with **(e)** representing Molecular Function and **(f)** representing KEGG Pathway analysis. Bottom x-axis represents the fold enrichment of genes in grey bars of interest over mouse genome background. Top x-axis represents –log_10_(FDR) by dot plot of genes of interest over mouse genome background. **(g)** Transcription factor binding sites enriched in the 99 overlapping regions from (b). p values calculated by Fisher exact test using HOMER.

To determine whether ACLY activity is responsible for histone acetylation at genes comprising the myofibroblast phenotype, we treated immortalized mouse CFs (with and without TGFβ) with the ACLYi and performed chromatin immunoprecipitation (ChIP) for H3K27ac, a histone mark on a lysine residue that we have previously shown to be demethylated in response to TGFβ stimulation^16,18^. ACLY inhibition was sufficient to prevent TGFβ-mediated enrichment of H3K27ac at fibrotic gene loci (*Acta2*, *Postn*) as assessed by ChIP–qPCR (Fig. 5a). To ascertain how ACLY influences global H3K27ac occupancy in cardiac myofibroblasts, primary CFs were treated with and without TGFβ, and with and without ACLYi, for 12 h and subjected to cleavage under targets and release using nuclease (CUT&RUN) for next-generation sequencing to determine ACLY-dependent H3K27ac occupancy. Principal component analysis of H3K27ac occupancy across the genome revealed tight clustering of the control groups, while TGFβ alone and TGFβ plus ACLYi groups clustered separately from the control and each other (Fig. 5b). Comparing differentially occupied regions between TGFβ alone and the control, we identified 3,475 regions that increased H3K27ac occupancy and 1,400 regions that decreased occupancy (Fig. 5c), reflecting the importance of histone acetylation and deacetylation in cardiac myofibroblast activation^35–38^. When comparing TGFβ plus ACLYi and control treatment regions, 1,818 regions increased and 2,652 regions decreased in H3K27ac occupancy (Fig. 5c). Comparing TGFβ plus ACLYi dual treatment with TGFβ alone revealed that only 3 regions increased and 601 regions decreased (Extended Data Fig. 6a). When comparing the control and ACLYi alone, we observed no significant changes in H3K27ac occupancy, suggesting that ACLY is not involved in the maintenance of H3K27ac occupancy in resting or non-activated CFs (Extended Data Fig. 6a). Next, we compared genes that showed increased H3K27ac occupancy with TGFβ stimulation alone versus the control with genes that showed increased H3K27ac occupancy with TGFβ plus ACLYi dual treatment compared with the control. This revealed loci that increased in H3K27ac occupancy following TGFβ treatment that were blocked by ACLY inactivation. This comparison revealed 172 regions that increased in the dual treatment, 1,646 regions that remained increased despite ACLYi treatment and 1,829 regions that were only increased in TGFβ alone, and thus were blocked by inhibition of ACLY (Fig. 5d). Examples of these ACLYi-sensitive regions can be viewed in Fig. 5e, which shows decreased occupancy of H3K27ac in promoter regions proximal to thrombospondin 1 (*Thbs1*) and hexokinase 2 (*Hk2*). We mapped these 1,829 regions to 253 unique genes (Supplementary Table 1). These ACLYi-sensitive regions are associated with several gene products important for myofibroblast differentiation, such as those involved in focal adhesions and stress fiber formation (*Actb*, *Actn1*, *Mfap4*), inflammatory and fibrotic paracrine signaling (*Lif*, *Tnfrsf12a*) and transcriptional regulation (*Foxs1*, *Wwtr1*, *Creb3l1*)^39–43^. Gene ontology enrichment analysis of these genes showed both biological processes and cellular components such as actomyosin, contractile actin filaments, and regulation of SMAD and TGFβ pathways (Fig. 5f,g). We selected a number of genes with ACLYi-dependent blockade of H3K27ac to confirm that their expression was decreased. Indeed, ACLYi decreased the TGFβ-induced expression of several H3K27ac-marked genes, such as *Loxl1*, *Tnfrsf12a* and *Serpine1* (Extended Data Fig. 4). Examples of H3K27ac-enriched loci sensitive to ACLYi can be viewed in Extended Data Fig. 5. Transcription factor motif enrichment analysis of ACLYi-dependent downregulated H3K27ac regions using Hypergeometric Optimization of Motif EnRichment (HOMER) identified the DNA-binding motifs for the AP-1 family of transcription factors, TEAD1 and SMAD2/3/4, all known mediators of canonical TGFβ signaling^44^ (Table 1 and Supplementary Data 1). Given the substantial number of genes containing SMAD2/3 binding motifs, we next tested whether SMADs physically interact with ACLY as a possible means of localizing it to specific genomic loci. Co-immunoprecipitation (co-IP) using specific SMAD2/3 and ACLY antibodies showed that ACLY was enriched with SMAD2/3 pulldown over background IgG, and vice versa, and this interaction was stabilized with TGFβ stimulation (Fig. 5h). Altogether, these data suggest that ACLY regulates H3K27ac occupancy at genes related to TGFβ-mediated myofibroblast identity and function, potentially by directly associating with SMAD2/3 for local supply of acetyl-CoA.

**Table 1:** Transcription factor motifs enriched in 1,829 ACLYi-sensitive regions

Motif	Name	*P* value	*q* value (Benjamini)	Targets with motif (%)
	AP-1	1.00 × 10^−35^	0.0000	14.60
	Smad4	1.00 × 10^−17^	0.0000	29.85
	Smad2	1.00 × 10^−13^	0.0000	27.88
	TEAD1	1.00 × 10^−11^	0.0000	12.47
	Smad3	1.00 × 10^−11^	0.0000	44.18

*P* values calculated by Fisher exact test using HOMER. AP-1, activator protein 1; TEAD1, TEA domain family member 1.

Using alternative parameters to analyze our CUT&RUN dataset, we defined ACLYi-sensitive H3K27ac occupancy as the regions that overlap between the 3,475 regions increased by TGFβ alone versus the control and the 601 regions decreased in TGFβ plus ACLYi versus TGFβ alone (Extended Data Fig. 6b). This discovered 99 overlapping regions that mapped to 89 unique genes that we used for gene ontology and HOMER transcription factor motif enrichment analyses. Gene ontology showed that the enrichment of genes involves stress fiber formation, focal adhesions and regulation of the SMAD pathway (Extended Data Fig. 6c,d and Supplementary Table 2), very similar to what was enriched in our previous analysis (Fig. 5g,h). ACLYi-sensitive genes from both analyses are reported in Supplementary Table 3. The 89 ACLYi-sensitive genes enriched for gene ontology for actin binding under molecular function and the Hippo signaling pathway by Kyoto Encyclopedia of Genes and Genomes (KEGG) pathway analysis (Extended Data Fig. 6e,f). Transcription factor motif analysis significantly enriched for Smad3 and Smad4 motifs (Extended Data Fig. 6g and Supplementary Data 2), showing again that ACLYi-sensitive H3K27ac regions are probably associated with SMAD signaling.

### ACLY inhibition reverts the myofibroblast phenotype of human CF

**Fig. 6:**
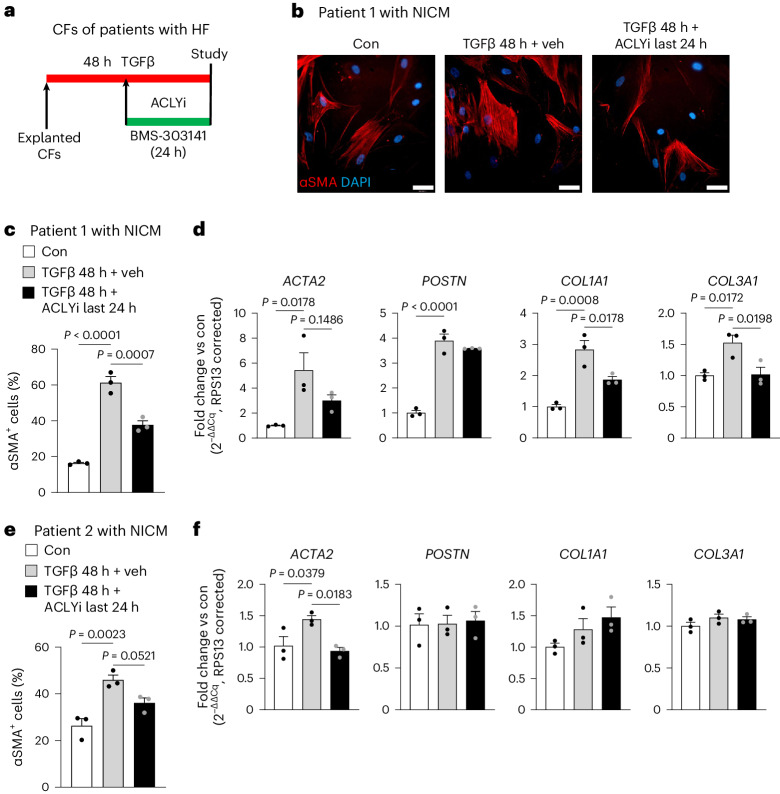
ACLY inhibition in human CFs from a patient with HF reverses myofibroblast fate and the fibrotic phenotype. **a**, Timeline of reversion experiments, human CFs isolated from NICM patients were treated with or without 4 μM BMS-303141 (ACLYi). **b**, Imaging of hCFs showing an αSMA-striated myofibroblast structure from patient 1 with NICM. Blue, DAPI-stained nuclei; red, αSMA. Scale bars, 50 μm. **c**, Quantification of αSMA^+^ myofibroblasts in patient 1 with HF. **d**, Myofibroblast gene expression using qPCR in CFs of patient 1 with HF. **e**, Quantification of αSMA^+^ myofibroblasts in patient 2 with HF. **f**, Myofibroblast gene expression using qPCR in CFs of patient 2 with HF. *n* = 3 technical replicates per group for each patient. Bars represent means, and error bars represent the s.e.m. Analysis was done using one-way ANOVA with Dunnett’s HSD. Source data

As the most therapeutically relevant aspect of targeting ACLY lies in the ability to dedifferentiate activated myofibroblasts to a less fibrotic phenotype, we tested the translational potential of ACLY-mediated myofibroblast reversion in human CFs isolated from HF donors diagnosed with non-ischemic cardiomyopathy (NICM). CFs isolated from failing human hearts were treated with and without TGFβ for 48 h with ACLY inhibition occurring at the 24 h mark (Fig. 6a), as previously outlined^15^, and the myofibroblast phenotype was determined by calculating the percentage of αSMA^+^ cells and expression of key fibrotic genes. Inhibition of ACLY reversed the myofibroblast phenotype, as indicated by a decrease in the percentage of αSMA^+^ myofibroblasts (Fig. 6b,c,e) and decreased expression of several fibrotic genes (Fig. 6d,f). While the responsiveness of human CFs to TGFβ or the ability of ACLYi to reverse the phenotype shows some degree of variability between donor hearts, the expression of the prototypical marker of a myofibroblast, αSMA, was conserved across all HF etiologies. In summary, these data indicate the therapeutic potential for ACLY inhibition to modulate the fibroblast phenotype at the level of histone acetylation, thereby providing an approach to prevent and reverse cardiac fibrosis.

## Discussion

Extensive cardiac myofibroblast differentiation and persistence are responsible for pathological fibrotic remodeling observed in nearly all etiologies of HF^3^. Attaining and maintaining the myofibroblast identity require metabolic alterations that we theorized in our previous work regulate chromatin remodeling and functional processes central to cellular fate^6,15,16,18^. The metabolic underpinnings of myofibroblast identity open a plethora of potential therapeutic solutions for cardiac fibrosis and HF. Here we show the impact of targeting ACLY in myofibroblast differentiation and cardiac fibrosis and provide a unique molecular mechanism that may govern loci-dependent acetylation to drive and maintain myofibroblast gene expression.

We discovered that not only does ACLY inactivation prevent myofibroblast differentiation in CFs, but also pharmacological ACLY inhibition is sufficient to revert the myofibroblast phenotype in mouse and human CFs under continuous fibrotic stimulation to a more quiescent or less fibrotic state. In mouse pressure overload, deletion of *Acly* in post-activated CFs preserved cardiac function and decreased interstitial cardiac fibrosis. These results show the potential utility of targeting ACLY in directed therapy to prevent or reverse fibrosis in HF. The ACLY inhibitor used in our study (BMS-303141) is orally available and cell membrane permeable but has a short in vivo half-life^30^. Pharmacologic targeting of ACLY could be achieved by modifying existing inhibitors yielding a relatively short pathway from bench to bedside. For instance, bempedoic acid is a first-in-class ACLY inhibitor for dyslipidemia that is an alternative to statins because it avoids conversion to its active form in skeletal muscle^45^. While the role of ACLY in cardiomyocytes is still unknown, this mechanism could be advantageous if bempedoic acid can inhibit ACLY in CFs while preserving its potential function in cardiac muscle. Alternatively, use of cell-type-specific vectors for delivery of antisense oligonucleotides against *ACLY* mRNA could circumvent current limitations and/or off-target effects of these drugs.

ACLY inhibition decreased H3K27ac occupancy at fibrotic genes that were significantly increased with TGFβ stimulation. Genes with decreased H3K27ac occupancy with inhibition of ACLYi in the context of pro-fibrotic agonist TGFβ are known regulators of the myofibroblast identity and function, such as *Col11a1*, *Loxl1*, *Mfap4* (ref. ^40^), *Tead4* (ref. ^46^) and *Serpine1* (ref. ^47^). The cellular components and biological processes identified by gene ontology enrichment indicate that ACLY regulates histone acetylation at genes related to the functional identity of the myofibroblast (stress fiber, smooth muscle migration, focal adhesion and so on) and signaling pathways (SMAD protein phosphorylation, epithelial-to-mesenchymal transition and so on). Identification of Hippo signaling from KEGG pathway analysis is noteworthy as the effectors and targets of this pathway have recently described roles in the myofibroblast fate^48^, suggesting that ACLY may also play a role in the signaling upstream of the Hippo pathway. Transcription factor motif analysis of the 1,829 ACLYi-sensitive regions identified the SMAD DNA-binding element as strongly enriched. To define what may be directing ACLY-dependent H3K27ac at these regions, we provide evidence of SMAD2/3 directly interacting with ACLY. These results suggest that ACLY is chaperoned by SMADs to ‘fibrotic’ genomic loci to supply HATs with acetyl-CoA for histone acetylation and maintenance of gene expression. This further implies that ACLY may form a ‘chrometabolon’ complex with SMAD and co-activators, such as p300, to facilitate histone acetylation at discrete genomic regions. In support of this hypothesis, a rapid immunoprecipitation mass spectrometry dataset identified p300 as an interactor with ACLY in chromatin^49^. Other data supporting our chrometabolon hypothesis include a report detailing p300 as a chaperone for SMAD2/3 nuclear importation^50^; thus, one can envision a p300–ACLY–SMAD2/3 complex mediating metabolite-dependent, sequence-specific histone acetylation to activate and lock in myofibroblast fate. Future studies aimed at identifying the exact protein–protein interactions of this complex may provide a therapeutic strategy to prevent the pro-fibrotic actions of ACLY while preserving physiological functions of ACLY, such as de novo lipid synthesis^51^.

Limitations of our work include being unable to directly resolve acetyl-CoA levels in the myofibroblast nucleus owing to technical limitations and to determine whether such supply is dependent on ACLY^52^. Given our CUT&RUN and ACLY co-IP data, even resolving nucleoplasm concentrations of acetyl-CoA may not fully appreciate the influence of ACLY on acetyl-CoA supply for histone acetylation if concentrations of such metabolites are regulated at the level of specific chromatin domains. One method to account for this limitation was our use of a nucleus-restricted, catalytically inactive ACLY construct that showed a dominant negative effect by blunting TGFβ-stimulated myofibroblast differentiation. This effect, together with ChIP and CUT&RUN data, allowed us to conclude that ACLY-derived acetyl-CoA synthesis in the nucleus regulates myofibroblast differentiation in a chromatin-dependent manner.

In summary, we establish ACLY activity as a primary mediator and maintainer of myofibroblast identity by directing H3K27ac occupancy at specific loci associated with the myofibroblast gene program. ACLY inactivation mitigated the myofibroblast phenotype and the underlying fibrotic gene program in multiple model systems. We underscore that nuclear localization of metabolic enzymes, such as ACLY, is a meaningful event in the physiological determination of cellular identity. Epigenetically, we reveal the unique function of ACLY to regulate discrete genomic loci associated with specific DNA-binding motifs, notably SMAD motifs, and SMAD2/3 directly associates with ACLY. Collectively, these data provide several therapeutic targets for treating HF by abating cardiac fibrosis. Inactivating ACLY directly or blocking its protein–protein interactions with chromatin and transcriptional complexes, such as SMAD2/3, could uncouple the metabolic–epigenetic crosstalk that maintains myofibroblast identity and cardiac fibrosis.

## Methods

### Animal work

All animal work complied with ethical regulations of animal testing and research in accordance with Institutional Animal Care & Use Committee approval by Temple University and followed Association for Assessment and Accreditation of Laboratory Animal Care guidelines. We used male and female C57BL/6NJ mice for all primary cell isolations and for all in vivo animal studies. *Acly*^fl/fl^ mice were created by Dr. Kathryn Wellen’s laboratory and purchased from Jackson Laboratories (MMRRC strain 043555-JAX)^29^. The mice were subsequently crossed with a mutant mouse model containing tamoxifen-inducible Cre-recombinase knocked into the *Postn* locus (*Postn*^iCre^; JAX stock 029645)^14^ to generate temporal control of *Acly* expression (*Acly*^fl/fl^, *Postn*^iCre^ line) exclusively in activated, post-differentiated CFs after injury. These mice were also crossed with mice expressing a *loxP*-flanked STOP cassette preventing transcription of a CAG promoter-driven red fluorescent protein variant (tdTomato), inserted into the *Gt(ROSA)26Sor* (*R26-tdT*) locus to allow for Cre-mediated fluorescent labeling of activated *Acly* knockout CFs (*Acly*^fl/fl^, *Postn*^iCre^, *R26-tdT*). All mice were generated on a C57BL/6NJ background. For in vivo studies, control mice had the following genotype: *Postn*^iCre^, *R26-tdT*. *Acly* knockout mice had the following genotype: *Acly*^fl/fl^, *Postn*^iCre^, *R26-tdT*. For temporal *Acly* deletion, male and female mice between the ages of 13 weeks and 15 weeks were randomized to TAC or sham cohorts and fed tamoxifen citrate chow (Envigo; 40 mg kg^−1^ d^−1^) starting the day following surgery and throughout the duration of the study. The first 2 weeks of tamoxifen diet had food pellets placed in a plastic dish and moistened with water to increase palatability, ensuring consumption. All mouse genotypes and controls received tamoxifen chow.

### TAC

TAC was performed as previously described^53^. Briefly, mice were anesthetized with inhalation of 3% isoflurane and intubated. A 7-0 nylon suture was tied around the transverse thoracic aorta together with a 27-gauge needle that was subsequently removed to generate a constriction with a defined diameter. Sham surgery was performed using the same procedure without tying of the suture around the aorta. The mice were then extubated, their chests closed and subcutaneous injections of 0.1 mg kg^−1^ buprenorphine were administered for 2 days after the surgery.

### Echocardiography

Transthoracic echocardiography of the left ventricle was performed as previously described^15^. Briefly, hair over the mouse thorax was removed using Nair (Church & Dwight) under anesthesia with 3% isoflurane. Echocardiograms were captured using a Vevo2100 Imaging System (VisualSonics). Body temperature was maintained (approximately 37 °C) using a heated stage and pre-warmed ultrasound gel and monitored using a rectal thermometer. Anesthesia was induced with 3% isoflurane, and the mice were maintained and examined with 1.5% isoflurane. Using long-axis B-modes and endocardial values, stroke volume and ejection fraction were calculated as previously described^54^. Long-axis B-modes were also used for the measurement of myocardial strain and strain rates using speckle tracking echocardiography. Using short-axis M-modes, left ventricular diameters during diastole (LVIDd), left ventricular diameters during systole (LVIDs), fractional shortening (FS%; LVIDd − LVIDs/LVIDd × 100) and heart rate were determined. Relative wall thickness was calculated as (diastolic posterior wall thickness + diastolic anterior wall thickness)/LVIDd. Color and pulse wave Doppler was used to calculate mean and peak aortic pressure gradients (mm Hg). To be included in TAC cohorts, banded mice were required to have a peak aortic pressure gradient surpassing 30 mm Hg. All analyses were performed using VisualSonics software. Experimentally blinded individuals performing echocardiography and image tracing were unaware of the genotypes and group assignments to prevent bias.

### Tissue gravimetrics

To assess differences in the cardiac hypertrophic response, heart weight to tibia length and heart weight to body weight ratios were calculated. Lung edema was determined by weighing the lungs at the time of isolation (wet lung mass) and again after dehydration at 37 °C for 2 days (dry lung mass) and subtracting the dry lung mass from the wet lung mass.

### Histology

Following euthanasia, excised hearts were flushed with 60 mM of KCl solution to depolarize the myocardium, fixed in 2% agarose in phosphate-buffered saline (PBS) and sectioned into 1 mm cross sections using the McIlwain Tissue Chopper. The fourth cross section from the apex was fixed in fresh 4% paraformaldehyde–PBS solution for 3 h at 4 °C, washed in PBS three times for 5 min each and then incubated in 30% sucrose solution in PBS overnight. The tissue was washed for 5 min in PBS and fixed in optimal cutting temperature compound using pre-cooled isopentane (2-methylbutane) as the freezing agent and stored at −80 °C; then, the tissue was sectioned at a thickness of 10 µm (Leica Cryotome CM1950), mounted on glass slides and stored at −80 °C until immunohistological approaches were performed.

To determine tissue fibrosis, the slides were stained for collagen content via sirius red staining (Abcam, catalog number ab150681). The slides were brought to room temperature, washed with PBS and incubated in Bouin’s solution for 1 h at 55 °C. The slides were then rinsed with double-distilled H_2_O and incubated in sirius red for 1 h at room temperature. The slides were then briefly rinsed in 0.5% acetic acid and dehydrated with xylene; Permount mounting medium was applied, and the slides were covered with coverslips. Images were acquired using a Nikon SMZ1000 microscope and analyzed using ImageJ software for measuring the percentage of fibrotic area. All images taken were limited to the left ventricle, and analysis did not include perivascular fibrosis. To prevent bias, the individuals performing staining, imaging and quantification were unaware of the genotypes and group assignments.

### Mouse CF isolation and culture

CFs were isolated from mice using a Langendorff perfusion apparatus. Mice were heparinized (200 U) for 15 min via intraperitoneal injection. Immediately after euthanasia, hearts were excised from mice and coronary arteries flushed with perfusion buffer (120 mM NaCl, 14.7 mM KCl, 0.6 mM KH_2_PO_4_, 0.6 mM Na_2_HPO_4_, 1.2 mM MgSO_4_, 10 mM Na-HEPES, 4.6 mM NaHCO_3_, 30 mM taurine, 10 mM 2,3-Butanedione monoxime (BDM), 5.5 mM glucose). The aorta was then cannulated and the hearts were perfused with 37 °C perfusion buffer for 5 min at a flow rate of 3.0 ml min^−1^ through the Langendorff apparatus. The hearts were then perfused with digestion buffer (perfusion buffer supplemented with 1 mg ml^−1^ collagenase B (Sigma; 11088807001), 140 μg ml^−1^ trypsin and 12.5 μM CaCl_2_) for 13 min. Atria and vasculature were then removed, and the ventricles mechanically dissociated with tweezers, scissors and pipetting. Dissociated ventricular tissue was then incubated in 5 ml of digestion buffer at 37 °C for 4 min before adding 5 ml of stop solution (perfusion buffer with 10% fetal bovine serum (FBS) and 12.5 μM CaCl_2_). Cardiomyocytes were pelleted by centrifugation at 30 × *g* for 3 min and discarded, and the resulting supernatant was centrifuged at 400 × *g* for 4 min to pellet the CFs. The CFs were plated in a 10 cm dish with Dulbecco’s modified Eagle’s medium (DMEM, Corning 10-013-CV) supplemented with 10% FBS (Peak Serum), 1% penicillin–streptomycin (Sigma) and 1% non-essential amino acids (Gibco), and incubated at 37 °C in the presence of 5% CO_2_. Following 1 h of incubation to allow for the rapidly adherent fibroblasts to attach, the medium was replaced to remove non-adherent cells and debris. Fresh medium was replaced daily until cells reached 80% confluence, at which time they were trypsinized and plated for downstream applications. All CFs for experiments were used at passage 1.

### Human CF isolation and culture

The use of human heart tissue in this study was approved by the institutional review board at the University of Pennsylvania. Written informed consent was obtained from the human donor providing explanted heart tissue for this study. The transplant recipients’ failing hearts received cold, blood-containing, high-potassium cardioplegic solution in vivo. Removed hearts were transported from the operating suite to the laboratory in cold Krebs–Henseleit buffer (KHB) solution (12.5 mM glucose, 5.4 mM KCl, 1 mM lactic acid, 1.2 mM MgSO_4_, 130 mM NaCl, 1.2 mM NaH_2_PO_4_, 25 mM NaHCO_3_ and 2 mM Na pyruvate, pH 7.4). Cardiac cell populations were disaggregated using modified isolation techniques previously described^55,56^. Briefly, hearts were weighed and rinsed in KHB. A non-infarcted free wall region of the LV apex was dissected, and a small catheter was placed into the lumen of the left anterior descending coronary artery. Major large vessels on the tissue piece were also ligated to improve perfusion. The temperature was maintained at 37 °C throughout the isolation. The cannulated LV tissue was perfused with a non-recirculating Ca^2+^-free solution (KHB containing 20 mM BDM and 10 mM taurine) for 10–15 min until the outflow temperature reached around 37 °C. Next, 200 ml of KHB containing 294 U ml^−1^ collagenase, 20 mM BDM and 10 mM taurine was perfused for 3 min without recirculation, followed by 22–32 min of recirculation. Ca^2+^ was introduced stepwise per minute by adding CaCl_2_ solution up to 1 mM, that is, 4 × 50 μM, 4 × 100 μM and 2 × 200 μM into the recirculated collagenase solution. Then, the tissue was perfused for 5 min with rinse solution (KHB containing 10 mM taurine, 20 mM BDM, 1 mM CaCl_2_ and 1% BSA). The tissue was then removed from the cannula, and myocardial tissue was minced in the rinse solution and triturated using glass pipets. The resulting cell suspension was filtered through 280 μm nylon mesh (Component Supply U-CMN-280), centrifuged (25 × *g* for 2 min) and resuspended in rinse solution. CFs were collected by allowing viable cardiomyocytes to pellet by gravity-sedimentation for 5 min, and the resulting supernatant was transferred to a fresh tube. The supernatant was then spun down at 400 × *g* for 4 min; resuspended in DMEM (Corning 10-013-CV) supplemented with 10% FBS (Gemini Bio-Products), 1% penicillin–streptomycin (Sigma), 1% ampicillin and 1% non-essential amino acids (Gibco); plated on a 10 cm dish; and incubated at 37 °C in the presence of 5% CO_2_. Following 3 h of incubation to allow for the rapidly adherent cell population to attach (that is, CFs), the medium was replaced. Fresh medium was replaced daily until cells reached 80% confluence, at which time they were trypsinized, further cultured or plated for downstream applications; ampicillin was removed at this step. All human-derived CF experiments were used at passages 1 and 2.

### MEF isolation and culture

MEFs were isolated from C57BL/6NJ (wild type) mice. Embryos were isolated from pregnant females at embryonic day 13.5. The embryos were decapitated, and all the red organs were removed. Tissue was minced and digested in 0.25% trypsin supplemented with DNase for 15 min at 37 °C in the presence of 5% CO_2_ and gently agitated by pipetting to dissociate cells every 5 min. Cells from each embryo were suspended in DMEM (Corning 10-013-CV) supplemented with 10% FBS (Gemini Bio-Products), 1% penicillin–streptomycin (Sigma) and 1% non-essential amino acids (Gibco), plated on a 10 cm dish and incubated at 37 °C in 5% CO_2_. The growth medium was replaced 3 h later to allow MEFs to attach and remove non-adherent cells and debris. All cells for experiments were used at passages 3 and 4.

### Myofibroblast differentiation

Myofibroblast differentiation was stimulated in vitro using 10 ng ml^−1^ recombinant mouse TGFβ (R&D Systems, 7666-MB-005). In all experiments, FBS in media was reduced to 1% 18 h before and during treatment with TGFβ.

### ChIP

Fibroblasts in 10 cm dishes were rinsed once with PBS and then fixed with 1% paraformaldehyde for 20 min at room temperature, followed by washing three times with PBS for 5 min per wash. Fibroblasts were then lysed in 1 ml ChIP lysis buffer (50 mM TRIS, 10 mM EDTA, 1% SDS, protease inhibitors added fresh) for 10 min on ice. Lysates were sonicated using a water sonicator (QSonica) at 50% power, for 20 min of ‘on’ time (15 s ‘on’ and 45 s ‘off’ per cycle) to shear chromatin to lengths between 200 and 500 bp. Sonicates were then centrifuged at 20,000 × *g* for 30 min at +4 °C. Supernatants of the sheared chromatin were diluted 1:10 with IP buffer (20 mM TRIS, 150 mM NaCl, 2 mM NaCl, 0.02% Triton X-100, protease inhibitors added fresh). Each IP uses 300 μl of the 1:10 dilution, and 30 μl of the 1:10 dilution was kept aside for the 10% input. DNA-bound protein was immunoprecipitated using 0.75 μg anti-H3K27ac (Abcam, ab4729) or 2 μg normal rabbit IgG (PeproTech, 500-P00) overnight at +4 °C with rotation. Precipitates then had 10 μl of magnetic protein A/G beads (Pierce, Thermo Fisher; 88802) added and incubated for 4 h at +4 °C with rotation. After four washes with four different wash buffers (wash buffer 1: 0.1% SDS, 0.1% NaDOC, 1% Triton X-100, 150 mM NaCl, 1 mM EDTA, 20 mM HEPES; wash buffer 2: 0.1% SDS, 0.1% NaDOC, 1% Triton X-100, 500 mM NaCl, 1 mM EDTA, 20 mM HEPES; wash buffer 3: 0.25 M LiCl, 0.5% NaDOC, 0.5% NP-40, 1 mM EDTA, 20 mM HEPES; wash buffer 4: 1 mM EDTA, 10 mM Tris–HCl (TE buffer)), cross-linking was reversed by resuspending beads in 120 μl elution buffer (1% SDS, 100 mM NaHCO_3_) and incubating beads overnight in 65 °C. De-cross-linked DNA was purified using a Qiaquick PCR purification kit (Qiagen, 28106) and eluted in 40 μl double-distilled H_2_O, and 2 μl was used for each qPCR. Amplification was performed using SYBR Green Master Mix (Applied Biosciences, A25918). Primers used are listed in Supplementary Table 1.

### CUT&RUN sequencing

Cultured CFs were lightly fixed by adding 37% formaldehyde directly to culture media to achieve the desired final concentration of 0.1% formaldehyde and incubated for 1 min. Fixation was quenched by adding glycine to a final concentration of 125 mM. CUT&RUN was performed according to the manufacturer’s instructions (EpiCypher, CUTANA ChIC/CUT&RUN Kit version 3, 14-1048) with some modifications for fixed cells. Modifications are described as follows: CUT&RUN wash buffer was modified by additionally preparing it with 0.05% SDS, 1% Triton X-100 and 1 μM Trichostatin A. Antibodies used were Histone H3K27ac Antibody, SNAP-ChIP Certified (EpiCypher, 13-0045) and CUTANA IgG Negative Control Antibody for CUT&RUN and CUT&Tag (EpiCypher, 13-0042). After CUT&RUN enriched DNA was obtained, cross-linking was reversed by adding 0.8 μl 10% SDS and 1 μl of 20 μg μl^−1^ proteinase K to each reaction (~83 μl) and DNA was incubated overnight at 55 °C in a thermal cycler, progressing with the remaining protocol the following day. Equal amounts of *Escherichia coli* DNA were spiked into each CUT&RUN sample at the end of the MNase reaction for experimental normalization. Libraries were prepared using a CUTANA CUT&RUN Library Prep Kit (EpiCypher, 14-1001) according to the manufacturer’s instructions. Illumina paired-end sequencing was performed by Novogene and ensured a minimum read depth of 3 million read pairs per sample. The H3K27ac CUT&RUN data were processed using CUT-RUNTools-2.0 (ref. ^57^), which executes adapter trimming, read alignment, duplicate marking, peak calling and blacklist filtering (see Supplementary Methods for an exact list of software dependencies and versions used to generate the Conda environment for the analysis). Briefly, we deployed the pipeline to perform adapter trimming on raw FASTQ files using Trimmomatic^58^ and to align reads to the mm10 genome at an 85–95% alignment rate using bowtie2 (3.6–12.9% alignment rate to spike-in genome for non-IgG samples; see Supplementary Table 4 for alignment rates of individual samples)^59^. After duplicate alignments were marked using Picard’s (https://broadinstitute.github.io/picard/) MarkDuplicates and duplicates were removed using Samtools^60^, peak calling was performed for each sample using the MACS2 (ref. ^61^) callpeak function with -q 0.01 as a parameter to retain only peaks passing our statistical threshold of *q* < 0.01. After blacklisted peaks were removed, DiffBind^62^ version 3.2.7 was used to generate a list of consensus peaks measured in at least three samples across the experiment (‘dba.count’) with ‘minOverlap’ = 3, and statistically test for differential binding between biological conditions. Peaks are annotated based on their position to the closest transcriptional start site. Normalization by spike-in *E. coli* DNA was used for viewing browser tracks. Deduped.bam files and blacklist filtered peak files were used as inputs for DiffBind, and differentially occupied peaks were classified as those passing false discovery rate (FDR) < 0.05. Principal component analysis was performed using the ‘dba.plotPCA()’ function of diffBind, and MA plots were generated using ‘dba.plotMA()’ with ‘bLoess = F’ and ‘bSmooth = F’. One sample from the TGFβ plus ACLYi group did not cluster with the other data points of the same group and was removed from downstream analysis. We deployed the findMotifsGenome.pl function of HOMER (v4.10)^63^ to determine known transcription factor motifs in the 1,829 ACLYi-sensitive regions, using the mm10 genome as background. Gene ontology was performed using the DAVID Functional Annotation Tool (v6.8; https://david.ncifcrf.gov/tools.jsp)^64^ using the list of 253 unique genes identified against a *Mus musculus* background. Annotated terms sought were KEGG pathway and the following GO terms: biological processes, cellular components and molecular function.

### Statistics and scientific rigor

Statistical parameters including the value of *n*; the definition of center, dispersion and precision measures (mean ± s.e.m.); and statistical significance are reported in the figures and legends. A *P* ≤ 0.05 was considered statistically significant for all datasets. In the figures, *P* values are provided to denote statistical significance as calculated by unpaired Student’s *t-*test (for direct comparisons). Multiple comparisons were assessed by one-way or two-way analysis of variance (ANOVA) followed by post hoc multiple-comparison tests as indicated in the figure legends (*P < 0.05; **P < 0.01; ***P < 0.001; ****P < 0.0001). Gene Ontology enrichment using the DAVID Functional Annotation Tool calculated one-sided *P* values using EASE scores (a modified version of Fisher exact test), and *q* values were calculated using the Bonferroni–Šidák *P* value, Benjamini and FDR. Terms with a *P* < 0.05 and a *q* < 0.05 by FDR are considered statistically significant. For HOMER motif analysis, *P* values were calculated using Fisher exact test, and values of *P* < 0.05 are considered significant. In vivo data from male and female mice were analyzed separately, and no sex differences were identified. Outliers were identified using Grubbs test with an alpha of 0.05. Identified outliers are included visually in graphs for data transparency (symbolized by ‘×’) but are not considered during statistical comparisons between groups. This analysis applies to Fig. 3h. Statistical analysis was performed using GraphPad Prism 10.2.0.

### Reporting summary

Further information on research design is available in the Nature Portfolio Reporting Summary linked to this article.

### Supplementary information

Supplementary Information Supplementary Methods and Data 1 and 2.

Reporting Summary

Supplementary Tables Supplementary Tables 1–4.

### Source data

Source Data Fig. 1 Full-length western blots.

Source Data Fig. 2 Full-length western blots.

Source Data Fig. 3 Full-length western blots.

Source Data Fig. 4 Full-length western blots.

Source Data Fig. 5 Full-length western blots.

Source Data Extended Data Fig. 3 Full-length western blots.

Source Data Figs. 1–6 Statistical source data.

## Data Availability

CUT&RUN sequencing data as fastq and bigWig files may be accessed from the NCBI GEO repository using accession number GSE232010. Source data are provided with this paper.

## Code availability

All bioinformatic code used in this study are publicly available. See ‘CUT&RUN Sequencing’ of Methods and Supplementary Information for directions on how to replicate the data processing.
