# Supplementary material for: Nuclear ATP-citrate lyase regulates chromatin-dependent activation and maintenance of the myofibroblast gene program
Source: Nat Cardiovasc Res. 2024 Jul 5;3(7):869–82. doi: 10.1038/s44161-024-00502-3 (PMC11358007; doi:10.1038/s44161-024-00502-3)
Supplement: Supplementary file 1 — Supplementary Methods and Data 1 and 2. [file 44161_2024_502_MOESM1_ESM.pdf]

nature cardiovascular research

Supplementary information https://doi.org/10.1038/s44161-024-00502-3

Nuclear ATP-citrate lyase regulates chromatin-dependent activation and maintenance of the myofibroblast gene program

In the format provided by the authors and unedited

Table of Contents:

Supplemental Methods

Supplemental Materials Table 1: qPCR Primers

Supplemental Materials Table 2: Western blot antibodies

Supplemental Data 1: HOMER Motif Analysis from Figure 5h

Supplemental Data 2: HOMER Motif Analysis from Extended Data Figure 6g

Supplemental Methods:

FACS. Injured hearts were excised and retrogradely perfused with 3mL ice-cold PBS and atria and vasculature were removed. The heart was diced and minces into very fine pieces and repeat-pipetted 20 times with a 2mL serological pipette in 5mL digestion buffer (0.1% Collagenase B, 2.4 U/mL Dispase II, 2.5 mM $CaCl_2$, 5.56 mM glucose, 138 mM NaCl, 5.4 mM KCl, 4.16 mM $mNaHCO_3$) for enzymatic dissociation. Digesting tissue incubated in 37°C for 30 min with additional repeat-pipetting to promote more physical dissociation. After 30 min enzymatic digestions were stopped by adding 5mL Stop Buffer (HBSS supplemented with 2% FBS, 1 mM HEPES) and passed through a 70-μm filter. The cell filtrate was centrifuged at 530 x g for 5 min at 4ºC, then the pellet resuspended in 5mL Stop Buffer, and passed through a 40-μm filter. The filtrate was centrifuged again at 530 x g for 5 min at 4ºC and the pellet resuspended in RBC Lysis Buffer (1.5M NH_4Cl, 100mM $NaHCO_3$, 10mM EDTA; pH 7.4) for 5 min. Finally, cells were then centrifuged at 530 x g for 5 min at 4ºC and the pellet resuspended in 1mL DMEM with 1% FBS. Cells were then sorted for TdTomato positivity using a BD FACS Aria-IIu cell sorter and collected for downstream analysis. To obtain enough cells, TdTomato+ sorted cells were pooled from multiple hearts.

Immortalized mouse CFs. Protocol for generating immortalized mouse CFs adapted from protocol in Alexanian *et al*. [56]. A *Tcf21*^{iCre}[57] mouse and a *R26-tdT* mouse were crossed to generate *Tcf21*^{iCre}*, R26-tdT*. Adult mice at 12 weeks of age were fed tamoxifen citrate chow (Envigo; 40mg/kg/day) for two weeks before FACS isolation of fluorescent cells as described above. About 40,000 tdTomato+ CFs were seeded into one well of a six-well plate and allowed to adhere and recover overnight in culture with Dulbecco's Modified Eagle's Medium (DMEM, Corning 10-013-CV) supplemented with 10% fetal bovine serum (FBS, Gemini Bio-Products), 1% penicillin/streptomycin (Sigma), and 1% Non-Essential Amino Acids (Gibco). Next day, media was replaced with Opti-MEM (ThermoScientific, 31985070) supplemented with 4μg/mL

polybrene for 1hr before infection with 2μL of SV40 T antigen expressing VSV-G pseudotyped lentiviral particles (Alstem, #CILV01). Infection proceeded for 24hr before virus-containing media was replaced with DMEM supplemented as described earlier in this section. After 2 days to recover from infection, infected CFs were cultured in media containing 1μg/mL puromycin for positive selection of infected cells for stable cell-line generation for 10 days, after which clones were picked for expansion. Culturing media conditions for immortalized CFs are the same as described above for MEFs and primary CFs. For sequential passages, immortalized CFs were split 1:10 and cultured to confluence in 2-3 days.

Adenoviral transfer. *Acly*$^{fl/fl}$ MEFs were trypsinized, counted by a hemocytometer, and seeded on 10-cm plates while allowing them to re-attach for at least 1hr. Growth media was then aspirated, cells rinsed once with PBS, and media replaced with Opti-MEM (ThermoScientific, 31985070). Samples were incubated with Opti-MEM for at least 1hr before adding adenovirus containing either Cre Recombinase (Vector Biolabs, 1045) or β-galactosidase (Vector Biolabs, 1080) as a control. Adenoviral infection proceeded for 24hr and MEFs cultured for 6d post infection to allow for sufficient ACLY turnover before beginning an experiment.

Electroporation. MEFs were trypsinized and resuspended to a concentration of $1x10^6$cells/mL in Ingenio Electroporation solution (Mirus Bio, MIR 50114). 250K MEFs were loaded into a 4mm cuvette with 5μg of plasmid DNA. Electroporation was performed with one square-wave pulse at 300V for 20ms. MEFs were then immediately seeded into 35-mm collagen coated dishes with DMEM supplemented with 1% FBS, 1% penicillin/streptomycin, and 1% Non-Essential Amino Acids and allowed to recover in incubation for 24 hrs before treatments.

qPCR mRNA analysis. RNA was isolated using the RNeasy Mini Kit (Qiagen, 74104) according to the manufacturer's protocol. RNA (1μg) was reverse transcribed into cDNA using the High-Capacity cDNA Reverse Transcription Kit (ThermoFisher, 4368814) according to the

manufacturer's protocol. Thermocycler conditions were as follows: 25ºC for 10 min, 37ºC for 2 hrs, 85ºC for 5 min. Quantification of cDNA was done SYBR Green Master Mix (Applied Biosciences; A25918) following the manufacturer's protocol. Cycling conditions were as follows: 95ºC for 10 min followed by 40 cycles of amplification (95ºC denaturation for 15 sec, 60ºC annealing for 30 sec, 72ºC extension for 30 sec), followed by the addition of melt curve conditions to ensure primer specificity. All samples were analyzed in duplicate and averaged. Ribosomal Protein S13 (*RPS13*) was used as a housekeeping gene. Fold change in mRNA expression was measured using the Comparative CT Method ($2^{-\Delta\Delta CT}$). Primer sequences are listed in Supplemental Materials Table 1.

Western Blotting. For whole cell protein isolation, samples were lysed using RIPA buffer supplemented with protease (Roche, 11836153001) and phosphatase inhibitors (Roche, 04906837001). Samples were lysed for 20 min in 4ºC followed by vortexing for 10 seconds and incubating on ice for a further 30 min. Samples were centrifuged at 13,000xg for 20 min and the supernatant was collected and used for analysis. Protein isolation optimized for collagen from cultured cells modified from a protocol from Hamanaka *et al* [66]. Briefly, cells were rinsed with PBS and lysed with 200μL of ice-cold Collagen Isolation Lysis Buffer (25mM HEPES, 150mM NaCl, 0.1% SDS, 1% Triton X-100, 0.5% sodium deoxycholate, 2mM EDTA, 2mM EGTA, with protease and phosphatase inhibitors) for 30 min on ice. Lysates were then sonicated at 20% power for 10 seconds and centrifuged for 20 min at 13,000 x g, +4°C. Supernatant collected in a new tube for analysis.

Protein concentrations were quantified using Bradford Protein Assay Kit (Bioworld, 20831001-1) and equal amounts of protein were run by gel electrophoresis on TRIS-glycine SDS gels. Gel separated protein were transferred to PVDF membranes (EMD Milipore, IPFL00010) and blocked for 1 hr in Blocking buffer for Fluorescent Western Blotting (Rockland, MB-070). Probing with primary antibodies with incubation overnight at 4ºC on a rocker. Membranes were

washed with TBS-T three times, 5 min per wash, and then incubated with fluorescent secondary antibody for 1 hr at room temperature. Membranes were then once again washed with TBS-T three times, 5 min per wash and imaged on a LICOR Odyssey scanner. See Supplementary Materials Table 2 for all antibodies used in this study. See Supplementary Figures for full length blots of all western blot appearing in this study.

Co-immunoprecipitation (co-IP). Fully confluent cells on a 15-cm dish were rinsed once with ice-cold PBS and crosslinked with 1mM of dithiobis(succinimidyl propionate) (DSP) for 30 min at room temperature. Samples were rinsed twice with PBS and then lysed with 500μL of Pierce IP Lysis Buffer (Thermo Scientific, 87787) supplemented with protease and phosphatase inhibitors. Samples were scraped and collected into 1.5mL tubes and incubated on ice for 5 min with intermittent vortexing. Samples were then centrifuged at 13,000 x g for 10 min at +4°C. Supernatants were transferred to a clean tube and protein concentrations were quantified. Each IP was performed with 500μg of protein and 2μg of pulldown antibody. Primary IPs were incubated overnight at +4°C with end-over-end rotation. Next day, 25μL of pre-washed Sera-Mag SpeedBeads Protein A/G Magnetic Particles (Cytiva, 17152104010150) were added to each IP and incubated for 4 hrs +4°C with end-over-end rotation. Bead-enriched samples were washed three times with TBST and eluted with ddH_2O and 5x SDS buffer for subsequent SDS-PAGE gel electrophoresis and Western blotting. Antibodies for co-IP used in this study: anti-ACLY (Abcam, ab40793), anti-Smad2/3 (BD Biosciences, 610842), anti-p300 (Santa Cruz, sc-32244), anti-mouse IgG (Santa Cruz, sc-2025).

Nuclear Protein Fractionation. Extraction of nuclear protein protocol was modified from Xuan *et al* [67]. Cells were removed from culture and rinsed once with PBS before being lysed with cytosolic fraction isolation buffer (25mM Tris, 0.5mM EDTA, 0.5mM EGTA, protease and phosphatase inhibitors). Lysates were incubated on ice for 10 min and centrifuged at 14,000 x g for 12 min at 4°C to isolate the cytosolic fraction as the supernatant. Pellets were resuspended

with Nuclear Isolation Buffer A (pH 7.9, 10mM HEPES, 1.5mM $MgCl_2$, 10mM KCl, 1mM DTT (add fresh), protease and phosphatase inhibitors (added fresh)), incubated on ice for 10 min followed by centrifugation at 2,000 x g for 15 min at 4°C. Supernatant discarded, pellet resuspended with Nuclear Isolation Buffer B (0.1% Triton X-100 + Nuclear Isolation Buffer A). Samples incubated on ice for 10 min followed by centrifugation at 2,000 x g for 15 min at 4°C. Supernatant discarded once more and pellets resuspended with Nuclear Isolation Buffer C (pH 7.9, 20mM HEPES, 25% glycerol, 420mM NaCl, 1.5mM $MgCl_2$ 0.2mM EDTA, 0.5mM DTT (add fresh), protease and phosphatase inhibitors (add fresh)). Samples kept on ice for 40 min with agitation by vortex every 10 min. Nuclear fraction collected at the supernatant isolated by centrifuging samples at 25,000 x g for 30 min at 4°C.

Immunofluorescence. Fibroblasts were seeded on 35mm collagen-coated glass bottom dishes (MatTel; P35GCol-1.5-10-C) at 50K cells for mouse CFs and MEFs and at 4000 human CFs seeded directly in the glass center of the dish. Fibroblasts were fixed for 15 min in 4% paraformaldehyde in PBS, permeabilized for 15 min with 0.15% Triton X-100 in PBS, and then blocked with 10% goat serum in PBS for 1 hr, all at room temperature. Fibroblasts were incubated with primary antibody αSMA (1:1000, Sigma-Aldrich, A2547) for 2 hrs at room temperature and secondary antibody goat anti-mouse Alexa Fluor 594 (1:1000, ThermoFisher, A-11005) for 45 min at 37ºC. Lastly, fibroblasts were incubated with Hoechst 33342 to define cell nuclei. Cells were images on a Carl Zeiss Axio Observer Z1 fluorescent microscope. Images acquired in red (590ex/617em) and blue (350ex/461em) channels. Quantification of images were analyzed as the percentage αSMA positive cells to the total cells (determined by number of nuclei) per field. More than 70 cells per dish were analyzed. Individuals quantifying αSMA positive cells were unaware of group assignments to prevent bias.

CUT&RUN Data Processing Software.

```
# This file may be used to create an environment using:
# $ conda create --name <env> --file <this file>
# platform: linux-64
_libgcc_mutex=0.1=conda_forge
_openmp_mutex=4.5=2_gnu
_r-mutex=1.0.1=anacondar_1
bedops=2.4.41=h9f5acd7_0
bedtools=2.30.0=h468198e_3
binutils_impl_linux-64=2.39=h6ceecb4_0
bowtie2=2.4.5=py36hb79b6da_4
bwidget=1.9.14=ha770c72_1
bzip2=1.0.8=h7f98852_4
c-ares=1.18.1=h7f98852_0
ca-certificates=2022.9.24=ha878542_0
cairo=1.16.0=ha61ee94_1014
certifi=2021.5.30=py36h5fab9bb_0
curl=7.85.0=h7bff187_0
cycler=0.11.0=pyhd8ed1ab_0
deeptools=3.5.1=py_0
deeptoolsintervals=0.1.9=py36h91eb985_4
expat=2.4.9=h27087fc_0
font-ttf-dejavu-sans-mono=2.37=hab24e00_0
font-ttf-inconsolata=3.000=h77eed37_0
font-ttf-source-code-pro=2.038=h77eed37_0
font-ttf-ubuntu=0.83=hab24e00_0
fontconfig=2.14.0=hc2a2eb6_1
fonts-conda-ecosystem=1=0
fonts-conda-forge=1=0
freetype=2.12.1=hca18f0e_0
fribidi=1.0.10=h36c2ea0_0
gcc_impl_linux-64=12.2.0=hcc96c02_18
gettext=0.21.1=h27087fc_0
gfortran_impl_linux-64=12.2.0=h55be85b_18
giflib=5.2.1=h36c2ea0_2
graphite2=1.3.13=h58526e2_1001
gsl=2.7=he838d99_0
gxx_impl_linux-64=12.2.0=hcc96c02_18
harfbuzz=5.3.0=h418a68e_0
htslib=1.16=h6bc39ce_0
icu=70.1=h27087fc_0
igraph=0.9.11=pypi_0
importlib-resources=5.4.0=pypi_0
joblib=1.1.1=pypi_0
jpeg=9e=h166bdaf_2
kernel-headers_linux-64=2.6.32=he073ed8_15
keyutils=1.6.1=h166bdaf_0
kiwisolver=1.3.1=py36h605e78d_1
krb5=1.19.3=h3790be6_0
lcms2=2.12=hddcbb42_0
ld_impl_linux-64=2.39=hc81fddc_0
```

leidenalg=0.8.10=pypi_0
lerc=4.0.0=h27087fc_0
libblas=3.9.0=16_linux64_openblas
libcblas=3.9.0=16_linux64_openblas
libcurl=7.85.0=h7bff187_0
libdeflate=1.13=h166bdaf_0
libedit=3.1.20191231=he28a2e2_2
libev=4.33=h516909a_1
libffi=3.4.2=h7f98852_5
libgcc-devel_linux-64=12.2.0=h3b97bd3_18
libgcc-ng=12.2.0=h65d4601_18
libgfortran-ng=12.2.0=h69a702a_18
libgfortran5=12.2.0=h337968e_18
libglib=2.74.0=h7a41b64_0
libgomp=12.2.0=h65d4601_18
libiconv=1.17=h166bdaf_0
liblapack=3.9.0=16_linux64_openblas
libnghttp2=1.47.0=hdcd2b5c_1
libnsl=2.0.0=h7f98852_0
libopenblas=0.3.21=pthreads_h78a6416_3
libpng=1.6.38=h753d276_0
libsanitizer=12.2.0=h46fd767_18
libsqlite=3.39.4=h753d276_0
libssh2=1.10.0=haa6b8db_3
libstdcxx-devel_linux-64=12.2.0=h3b97bd3_18
libstdcxx-ng=12.2.0=h46fd767_18
libtiff=4.4.0=h0e0dad5_3
libuuid=2.32.1=h7f98852_1000
libwebp=1.2.4=h522a892_0
libwebp-base=1.2.4=h166bdaf_0
libxcb=1.13=h7f98852_1004
libxml2=2.9.14=h22db469_4
libzlib=1.2.13=h166bdaf_4
llvmlite=0.36.0=pypi_0
macs2=2.2.7.1=py36h91eb985_5
make=4.3=hd18ef5c_1
matplotlib-base=3.3.4=py36hd391965_0
ncurses=6.3=h27087fc_1
numba=0.53.1=pypi_0
numpy=1.19.5=py36hfc0c790_2
olefile=0.46=pyh9f0ad1d_1
openssl=1.1.1q=h166bdaf_0
pango=1.50.11=h382ae3d_0
parallel=20220922=ha770c72_0
pcre2=10.37=hc3806b6_1
perl=5.32.1=2_h7f98852_perl5
pillow=8.3.1=py36h5aabda8_0
pip=21.3.1=pyhd8ed1ab_0
pixman=0.40.0=h36c2ea0_0
plotly=5.10.0=pyhd8ed1ab_0
pthread-stubs=0.4=h36c2ea0_1001

py2bit=0.3.0=py36h91eb985_6
pybigwig=0.3.18=py36h54a71a5_2
pynndescent=0.5.7=pypi_0
pyparsing=3.0.9=pyhd8ed1ab_0
pysam=0.19.1=py36h50b03f4_1
python=3.6.15=hb7a2778_0_cpython
python-dateutil=2.8.2=pyhd8ed1ab_0
python_abi=3.6=2_cp36m
r-base=4.2.1=h7880091_2
readline=8.1.2=h0f457ee_0
samtools=1.16.1=h6899075_1
scikit-learn=0.24.2=pypi_0
scipy=1.5.3=py36h81d768a_1
sed=4.8=he412f7d_0
setuptools=58.0.4=py36h5fab9bb_2
six=1.16.0=pyh6c4a22f_0
sqlite=3.39.4=h4ff8645_0
sysroot_linux-64=2.12=he073ed8_15
tabix=1.11=hdfd78af_0
tbb=2021.6.0=h924138e_0
tenacity=8.1.0=pyhd8ed1ab_0
texttable=1.6.4=pypi_0
threadpoolctl=3.1.0=pypi_0
tk=8.6.12=h27826a3_0
tktable=2.10=hb7b940f_3
tornado=6.1=py36h8f6f2f9_1
tqdm=4.64.1=pypi_0
umap-learn=0.5.3=pypi_0
wheel=0.37.1=pyhd8ed1ab_0
xorg-kbproto=1.0.7=h7f98852_1002
xorg-libice=1.0.10=h7f98852_0
xorg-libsm=1.2.3=hd9c2040_1000
xorg-libx11=1.7.2=h7f98852_0
xorg-libxau=1.0.9=h7f98852_0
xorg-libxdmcp=1.1.3=h7f98852_0
xorg-libxext=1.3.4=h7f98852_1
xorg-libxrender=0.9.10=h7f98852_1003
xorg-libxt=1.2.1=h7f98852_2
xorg-renderproto=0.11.1=h7f98852_1002
xorg-xextproto=7.3.0=h7f98852_1002
xorg-xproto=7.0.31=h7f98852_1007
xz=5.2.6=h166bdaf_0
zipp=3.6.0=pypi_0
zlib=1.2.13=h166bdaf_4
zstd=1.5.2=h6239696_4

Supplemental Materials Table 1: qPCR Primers

Mouse primers for cDNA-qPCR	5' → 3'
Mouse_Rps13_Forward	GCACCTTGAGAGGAACAGAA
Mouse_Rps13_Reverse	GAGCACCCGCTTAGTCTTATAG
Mouse_Acta2_Forward	GTGAAGAGGAAGACAGCACAG
Mouse_Acta2_Reverse	GCCCATTCCAACCATTACTCC
Mouse_Postn_Forward	CCATTGGAGGCAAACAACTCC
Mouse_Postn_Reverse	TTGCTTCCTCTCACCATGCA
Mouse_Col1a1_Forward	TTCAGGGAATGCCTGGTGAA
Mouse_Col1a1_Reverse	ACCTTTGGGACCAGCATCA
Mouse_Col3a1_Forward	TGCTGGAAAGAATGGGGAGAC
Mouse_Col3a1_Reverse	GGTCCAGAATCTCCCTTGTCAC
Mouse_Fn1_Forward	CGTCATTGCCCTGAAGAACA
Mouse_Fn1_Reverse	AAGGGTAACCAGTTGGGGAA
Mouse_Pdgfra_Forward	CAAAGGGAGGACGTTCAAGAC
Mouse_Pdgfra_Reverse	TGCGTCCATCTCCAGATTCA
Mouse_Acly_Forward	TTCGTCAAACAGCACTTCC
Mouse_Acly_Reverse	ATTTGGCTTCTTGGAGGTG
Human primers for cDNA-qPCR	5' → 3'
Human_Rps13_Reverse	GCATCCTTATCCTTTCTGTTCCTCTC
Human_Acta2_Forward	CTTCGTTACTACTGCTGAGCGTGAG
Human_Acta2_Reverse	GGGGCAATGATCTTGATCTTCATGG
Human_Postn_Forward	CAACTTGGATTCTGATATCCGTAGAGG
Human_Postn_Reverse	GGTACTTCATAAGAGCTTCGGAAGCC

Human_Col1a1_Forward	TTCAGGGAATGCCTGGTGAA
Human_Col1a1_Reverse	ACCTTTGGGACCAGCATCA
Human_Col3a1_Forward	CAGGAGAAAAGGGTCCTGCTGG
Human_Col3a1_Reverse	CAGCATCACCCTTGCCTCCTG
Human_Fn1_Forward	GCCAAGAGACAGCTGTAACCCAG
Human_Fn1_Reverse	CAATGCACTGATCTCGAAGCTGC
Human_Pdgfra_Forward	GACTAGTGCTTGGTCGGGTCTTG
Human_Pdgfra_Reverse	CACGGTGGACACAATTTTTTGAAGCC
Human_Acly_Forward	ATCGGTTCAAGTATGCTCGGG
Human_Acly_Reverse	GACCAAGTTTTCCACGACGTT
Mouse Primers for ChIP-qPCR	5’ → 3’
ChIP_Mouse_Actb_Forward	TCTTGCCTAAATCCAGCTTTCTGT
ChIP_Mouse_Actb_Reverse	CCCCTCACCTAAGTACCAGTGT
ChIP_Mouse_NeuroD1_Forward	TTACCTTTCCAGCTCGCCTC
ChIP_Mouse_ NeuroD1_Reverse	ACTCCGCGGACCTGTTTTTA
ChIP_Mouse_Acta2_Forward	ATAGCAATGCCACCAGCTAGTTT
ChIP_Mouse_Acta2_Reverse	GGGCCTGTCCAAAATAAGCTTTC
ChIP_Mouse_Postn_Forward	CCACAGCCCAGAGACTATATAAAC
ChIP_Mouse_ Postn_Reverse	CAGCAGCAGCAGAGCATATAA

Supplemental Materials Table 2: Western blot antibodies

Target	Catalog Number	Dilution
Phospho ATP Citrate Lyase (Ser455) Antibody	Cell Signaling Technology 4331S	1:500
ATP-Citrate Lyase	Abcam Ab40793	1:1000
Alpha-Tubulin	Abcam Ab7291	1:1000
Collagen type I alpha-1	Cell Signaling Technology 72026S	1:500
Lamin B1	Abcam Ab229025	1:1000
Fibrillarin	Cell Signaling Technology 2639S	1:1000
Fatty Acid Synthase	Cell Signaling Technology 3189S	1:1000
GFP	Rockland 600-101-215M	1:1000
HDAC1	Cell Signaling Technology 5356T	1:1000
POSTN	Abcam Ab14041	1:1000
LDHA	Cell Signaling Technology 2012S	1:1000

Supplemental Data 1: HOMER Motif Analysis from Figure 5h. Homer Known Motif Enrichment Results (/media/tvlab/tb/2023_Elrod_H3K27ac/Figures/2023-02-13/2023-02-13_up_in_TGFb_only_1829_regions)

Homer *de novo* Motif Results
Gene Ontology Enrichment Results
Known Motif Enrichment Results (txt file)
Total Target Sequences = 1828, Total Background Sequences = 47509

Rank	Motif	Name	P-value	log P-pvalue	q-value (Benjamini)	# Target Sequences with Motif	% of Targets Sequences with Motif	# Background Sequences with Motif	% of Background Sequences with Motif	Motif File	SVG
1	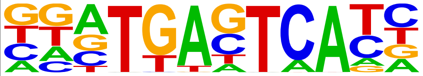	Fos(bZIP)/TSC-Fos-ChIP-Seq(GSE110950)/Homer	1e-49	-1.150e+02	0.0000	243.0	13.29%	2118.9	4.46%	motif file (matrix)	svg
2	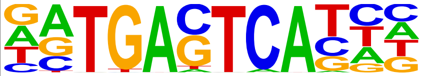	Atf3(bZIP)/GBM-ATF3-ChIP-Seq(GSE33912)/Homer	1e-44	-1.036e+02	0.0000	258.0	14.11%	2503.5	5.27%	motif file (matrix)	svg
3	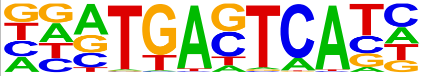	Fra1(bZIP)/BT549-Fra1-ChIP-Seq(GSE46166)/Homer	1e-44	-1.028e+02	0.0000	227.0	12.41%	2033.5	4.28%	motif file (matrix)	svg
4	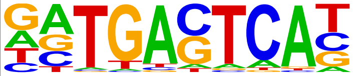	JunB(bZIP)/DendriticCells-Junb-ChIP-Seq(GSE36099)/Homer	1e-39	-9.087e+01	0.0000	223.0	12.19%	2135.2	4.49%	motif file (matrix)	svg
5	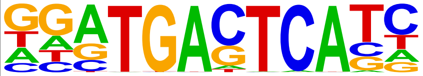	Fra2(bZIP)/Striatum-Fra2-ChIP-Seq(GSE43429)/Homer	1e-39	-9.017e+01	0.0000	206.0	11.26%	1882.7	3.96%	motif file (matrix)	svg
6	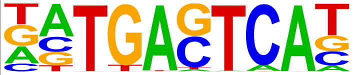	BATF(bZIP)/Th17-BATF-ChIP-Seq(GSE39756)/Homer	1e-38	-8.901e+01	0.0000	247.0	13.50%	2549.0	5.36%	motif file (matrix)	svg
7	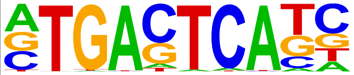	AP-1(bZIP)/ThioMac-PU.1-ChIP-Seq(GSE21512)/Homer	1e-35	-8.193e+01	0.0000	267.0	14.60%	3009.2	6.33%	motif file (matrix)	svg
8	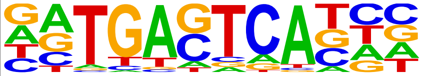	Fosl2(bZIP)/3T3L1-Fosl2-ChIP-Seq(GSE56872)/Homer	1e-28	-6.452e+01	0.0000	155.0	8.47%	1457.6	3.07%	motif file (matrix)	svg
9	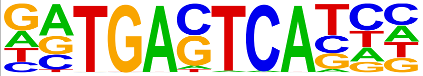	Jun-AP1(bZIP)/K562-cJun-ChIP-Seq(GSE31477)/Homer	1e-24	-5.558e+01	0.0000	118.0	6.45%	1023.1	2.15%	motif file (matrix)	svg
10	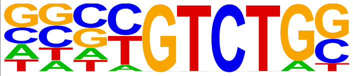	Smad4(MAD)/ESC-SMAD4-ChIP-Seq(GSE29422)/Homer	1e-17	-4.037e+01	0.0000	546.0	29.85%	10089.8	21.22%	motif file (matrix)	svg
11	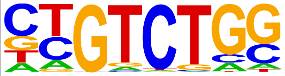	Smad2(MAD)/ES-SMAD2-ChIP-Seq(GSE29422)/Homer	1e-13	-3.196e+01	0.0000	510.0	27.88%	9699.4	20.40%	motif file (matrix)	svg
12	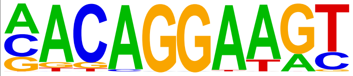	Ets1-distal(ETS)/CD4+-PolII-ChIP-Seq(Barski_et_al.)/Homer	1e-12	-2.973e+01	0.0000	122.0	6.67%	1527.3	3.21%	motif file (matrix)	svg
13	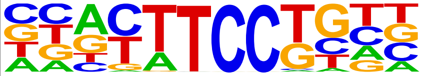	Etv2(ETS)/ES-ER71-ChIP-Seq(GSE59402)/Homer	1e-12	-2.802e+01	0.0000	282.0	15.42%	4787.5	10.07%	motif file (matrix)	svg

14	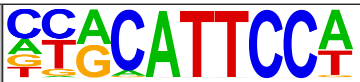	TEAD1(TEAD)/HepG2-TEAD1-ChIP-Seq(Encode)/Homer	1e-11	-2.744e+01	0.0000	233.0	12.74%	3774.1	7.94%	motif file (matrix)	svg
15	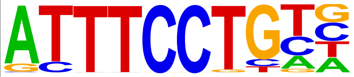	EWS:ERG-fusion(ETS)/CADO_ES1-EWS:ERG-ChIP-Seq(SRA014231)/Homer	1e-11	-2.689e+01	0.0000	199.0	10.88%	3094.6	6.51%	motif file (matrix)	svg
16	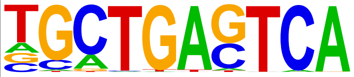	Bach2(bZIP)/OCILy7-Bach2-ChIP-Seq(GSE44420)/Homer	1e-11	-2.583e+01	0.0000	80.0	4.37%	880.3	1.85%	motif file (matrix)	svg
17	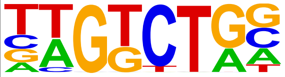	Smad3(MAD)/NPC-Smad3-ChIP-Seq(GSE36673)/Homer	1e-11	-2.535e+01	0.0000	808.0	44.18%	17357.4	36.51%	motif file (matrix)	svg
18	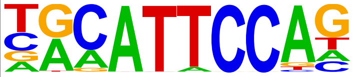	TEAD3(TEA)/HepG2-TEAD3-ChIP-Seq(Encode)/Homer	1e-9	-2.148e+01	0.0000	247.0	13.50%	4329.5	9.11%	motif file (matrix)	svg
19	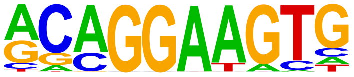	ETS1(ETS)/Jurkat-ETS1-ChIP-Seq(GSE17954)/Homer	1e-8	-2.071e+01	0.0000	301.0	16.46%	5560.2	11.69%	motif file (matrix)	svg
20	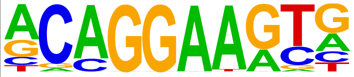	ERG(ETS)/VCaP-ERG-ChIP-Seq(GSE14097)/Homer	1e-8	-2.039e+01	0.0000	432.0	23.62%	8586.4	18.06%	motif file (matrix)	svg
21	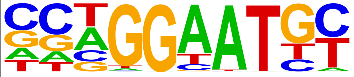	TEAD4(TEA)/Tropoblast-Tead4-ChIP-Seq(GSE37350)/Homer	1e-8	-1.964e+01	0.0000	205.0	11.21%	3510.9	7.38%	motif file (matrix)	svg
22	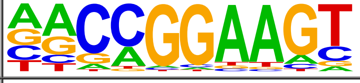	GABPA(ETS)/Jurkat-GABPa-ChIP-Seq(GSE17954)/Homer	1e-8	-1.932e+01	0.0000	267.0	14.60%	4878.7	10.26%	motif file (matrix)	svg
23	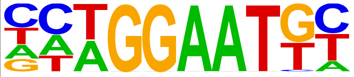	TEAD(TEA)/Fibroblast-PU.1-ChIP-Seq(Unpublished)/Homer	1e-8	-1.894e+01	0.0000	148.0	8.09%	2346.8	4.94%	motif file (matrix)	svg
24	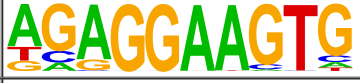	PU.1(ETS)/ThioMac-PU.1-ChIP-Seq(GSE21512)/Homer	1e-7	-1.828e+01	0.0000	160.0	8.75%	2619.1	5.51%	motif file (matrix)	svg
25	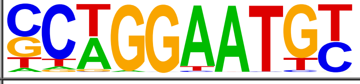	TEAD2(TEA)/Py2T-Tead2-ChIP-Seq(GSE55709)/Homer	1e-6	-1.610e+01	0.0000	129.0	7.05%	2066.6	4.35%	motif file (matrix)	svg
26	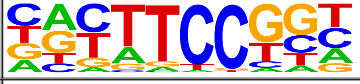	Fli1(ETS)/CD8-FLI-ChIP-Seq(GSE20898)/Homer	1e-6	-1.569e+01	0.0000	295.0	16.13%	5725.1	12.04%	motif file (matrix)	svg
27	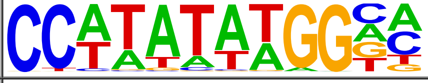	CArG(MADS)/PUER-Srf-ChIP-Seq(Sullivan_et_al.)/Homer	1e-6	-1.542e+01	0.0000	80.0	4.37%	1118.1	2.35%	motif file (matrix)	svg
28	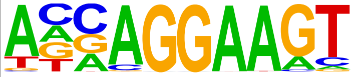	ELF3(ETS)/PDAC-ELF3-ChIP-Seq(GSE64557)/Homer	1e-6	-1.495e+01	0.0000	197.0	10.77%	3567.8	7.50%	motif file (matrix)	svg
29	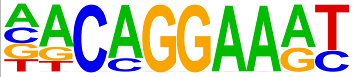	EWS:FLI1-fusion(ETS)/SK_N_MC-EWS:FLI1-ChIP-Seq(SRA014231)/Homer	1e-6	-1.418e+01	0.0000	164.0	8.97%	2888.3	6.07%	motif file (matrix)	svg
30	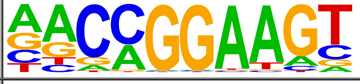	ETV1(ETS)/GIST48-ETV1-ChIP-Seq(GSE22441)/Homer	1e-6	-1.396e+01	0.0000	371.0	20.28%	7621.0	16.03%	motif file (matrix)	svg
31	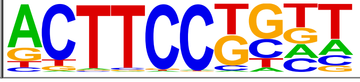	Elf4(ETS)/BMDM-Elf4-ChIP-Seq(GSE88699)/Homer	1e-5	-1.340e+01	0.0000	286.0	15.64%	5674.6	11.93%	motif file (matrix)	svg

32	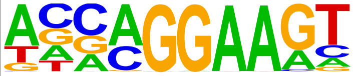	EHF(ETS)/LoVo-EHF-ChIP-Seq(GSE49402)/Homer	1e-5	-1.244e+01	0.0001	320.0	17.50%	6538.4	13.75%	motif file (matrix)	svg
33	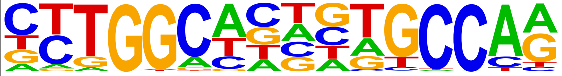	NF1(CTF)/LNCAP-NF1-ChIP-Seq(Unpublished)/Homer	1e-4	-1.142e+01	0.0001	120.0	6.56%	2078.3	4.37%	motif file (matrix)	svg
34	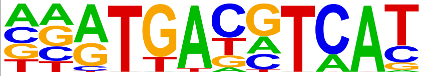	CREB5(bZIP)/LNCaP-CREB5.V5-ChIP-Seq(GSE137775)/Homer	1e-4	-1.050e+01	0.0004	79.0	4.32%	1262.0	2.65%	motif file (matrix)	svg
35	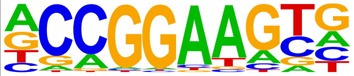	ETV4(ETS)/HepG2-ETV4-ChIP-Seq(ENCODE)/Homer	1e-4	-1.023e+01	0.0005	301.0	16.46%	6273.4	13.19%	motif file (matrix)	svg
36	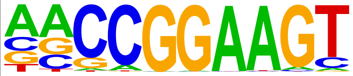	ETS(ETS)/Promoter/Homer	1e-4	-9.513e+00	0.0009	98.0	5.36%	1700.9	3.58%	motif file (matrix)	svg
37	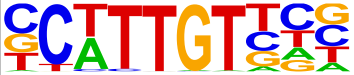	Sox10(HMG)/SciaticNerve-Sox3-ChIP-Seq(GSE35132)/Homer	1e-4	-9.215e+00	0.0012	320.0	17.50%	6818.9	14.34%	motif file (matrix)	svg
38	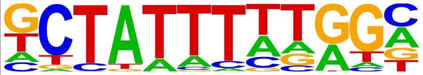	Mef2b(MADS)/HEK293-Mef2b.V5-ChIP-Seq(GSE67450)/Homer	1e-3	-9.046e+00	0.0014	115.0	6.29%	2091.7	4.40%	motif file (matrix)	svg
39	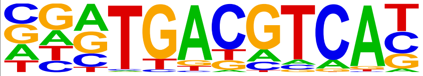	Atf7(bZIP)/3T3L1-Atf7-ChIP-Seq(GSE56872)/Homer	1e-3	-8.343e+00	0.0027	105.0	5.74%	1912.0	4.02%	motif file (matrix)	svg
40	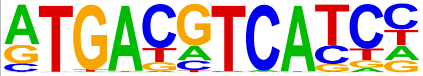	c-Jun-CRE(bZIP)/K562-cJun-ChIP-Seq(GSE31477)/Homer	1e-3	-8.062e+00	0.0035	71.0	3.88%	1196.8	2.52%	motif file (matrix)	svg
41	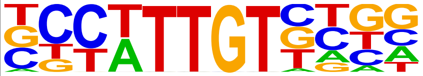	Sox21(HMG)/ESC-SOX21-ChIP-Seq(GSE110505)/Homer	1e-3	-7.952e+00	0.0038	330.0	18.04%	7186.6	15.11%	motif file (matrix)	svg
42	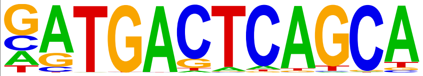	NF-E2(bZIP)/K562-NFE2-ChIP-Seq(GSE31477)/Homer	1e-3	-7.914e+00	0.0038	22.0	1.20%	248.6	0.52%	motif file (matrix)	svg
43	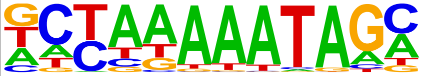	Mef2c(MADS)/GM12878-Mef2c-ChIP-Seq(GSE32465)/Homer	1e-3	-7.814e+00	0.0041	51.0	2.79%	795.6	1.67%	motif file (matrix)	svg
44	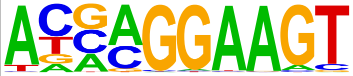	ELF5(ETS)/T47D-ELF5-ChIP-Seq(GSE30407)/Homer	1e-3	-7.076e+00	0.0085	177.0	9.68%	3630.7	7.64%	motif file (matrix)	svg
45	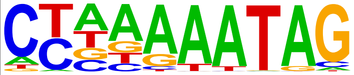	Mef2a(MADS)/HL1-Mef2a.biotin-ChIP-Seq(GSE21529)/Homer	1e-3	-6.940e+00	0.0095	58.0	3.17%	973.1	2.05%	motif file (matrix)	svg
46	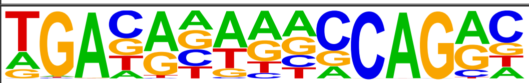	Hand2(bHLH)/Mesoderm-Hand2-ChIP-Seq(GSE61475)/Homer	1e-3	-6.934e+00	0.0095	139.0	7.60%	2763.0	5.81%	motif file (matrix)	svg
47	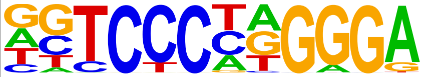	EBF(EBF)/proBcell-EBF-ChIP-Seq(GSE21978)/Homer	1e-2	-6.788e+00	0.0106	63.0	3.44%	1085.2	2.28%	motif file (matrix)	svg
48	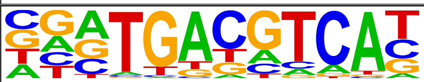	Atf2(bZIP)/3T3L1-Atf2-ChIP-Seq(GSE56872)/Homer	1e-2	-6.786e+00	0.0106	75.0	4.10%	1342.2	2.82%	motif file (matrix)	svg
49	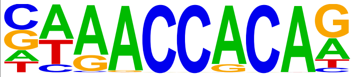	RUNX(Runt)/HPC7-Runx1-ChIP-Seq(GSE22178)/Homer	1e-2	-6.782e+00	0.0106	165.0	9.02%	3375.2	7.10%	motif file (matrix)	svg

50	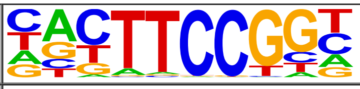	Elk1(ETS)/Hela-Elk1-ChIP-Seq(GSE31477)/Homer	1e-2	-6.719e+00	0.0106	144.0	7.87%	2894.3	6.09%	motif file (matrix)	svg
51	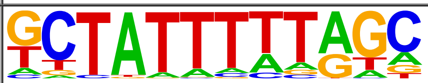	Mef2d(MADS)/Retina-Mef2d-ChIP-Seq(GSE61391)/Homer	1e-2	-6.649e+00	0.0112	25.0	1.37%	328.5	0.69%	motif file (matrix)	svg
52	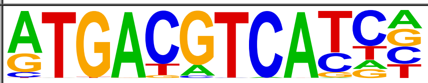	JunD(bZIP)/K562-JunD-ChIP-Seq/Homer	1e-2	-6.624e+00	0.0112	26.0	1.42%	347.9	0.73%	motif file (matrix)	svg
53	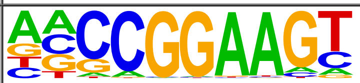	ELF1(ETS)/Jurkat-ELF1-ChIP-Seq(SRA014231)/Homer	1e-2	-6.615e+00	0.0112	136.0	7.44%	2718.3	5.72%	motif file (matrix)	svg
54	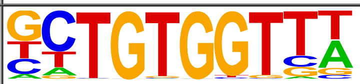	RUNX-AML(Runt)/CD4+-PolII-ChIP-Seq(Barski_et_al.)/Homer	1e-2	-6.468e+00	0.0126	161.0	8.80%	3308.2	6.96%	motif file (matrix)	svg
55	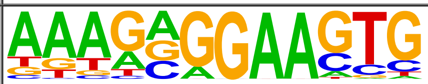	SpiB(ETS)/OCILY3-SPIB-ChIP-Seq(GSE56857)/Homer	1e-2	-6.445e+00	0.0127	58.0	3.17%	995.4	2.09%	motif file (matrix)	svg
56	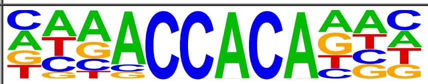	RUNX2(Runt)/PCa-RUNX2-ChIP-Seq(GSE33889)/Homer	1e-2	-6.287e+00	0.0146	182.0	9.95%	3816.5	8.03%	motif file (matrix)	svg
57	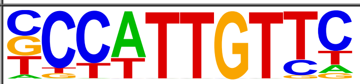	Sox2(HMG)/mES-Sox2-ChIP-Seq(GSE11431)/Homer	1e-2	-6.067e+00	0.0179	160.0	8.75%	3319.2	6.98%	motif file (matrix)	svg
58	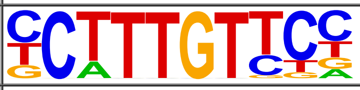	Sox4(HMG)/proB-Sox4-ChIP-Seq(GSE50066)/Homer	1e-2	-5.571e+00	0.0289	164.0	8.97%	3458.8	7.27%	motif file (matrix)	svg
59	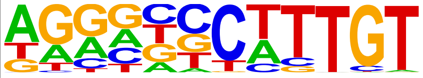	Sox9(HMG)/Limb-SOX9-ChIP-Seq(GSE73225)/Homer	1e-2	-5.528e+00	0.0296	184.0	10.06%	3936.8	8.28%	motif file (matrix)	svg
60	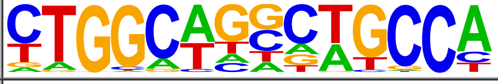	Tlx?(NR)/NPC-H3K4me1-ChIP-Seq(GSE16256)/Homer	1e-2	-5.460e+00	0.0312	126.0	6.89%	2578.8	5.42%	motif file (matrix)	svg
61	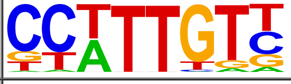	Sox3(HMG)/NPC-Sox3-ChIP-Seq(GSE33059)/Homer	1e-2	-5.459e+00	0.0312	315.0	17.22%	7115.1	14.96%	motif file (matrix)	svg
62	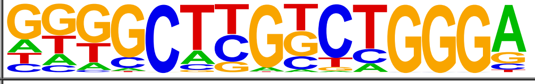	Zfp809(Zf)/ES-Zfp809-ChIP-Seq(GSE70799)/Homer	1e-2	-5.359e+00	0.0334	59.0	3.23%	1070.9	2.25%	motif file (matrix)	svg
63	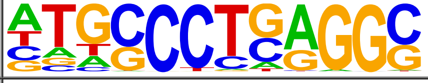	AP-2alpha(AP2)/Hela-AP2alpha-ChIP-Seq(GSE31477)/Homer	1e-2	-5.325e+00	0.0340	208.0	11.37%	4531.6	9.53%	motif file (matrix)	svg
64	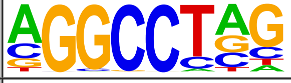	ZNF711(Zf)/SHSY5Y-ZNF711-ChIP-Seq(GSE20673)/Homer	1e-2	-5.270e+00	0.0354	471.0	25.75%	11015.7	23.17%	motif file (matrix)	svg
65	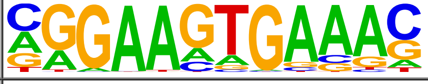	PU.1-IRF(ETS:IRF)/Bcell-PU.1-ChIP-Seq(GSE21512)/Homer	1e-2	-5.173e+00	0.0384	282.0	15.42%	6344.3	13.34%	motif file (matrix)	svg
66	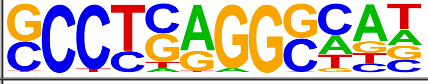	AP-2gamma(AP2)/MCF7-TFAP2C-ChIP-Seq(GSE21234)/Homer	1e-2	-5.115e+00	0.0400	272.0	14.87%	6107.6	12.85%	motif file (matrix)	svg
67	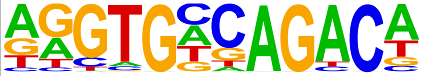	Tbox:Smad(T-box,MAD)/ESCd5-Smad2_3-ChIP-Seq(GSE29422)/Homer	1e-2	-5.047e+00	0.0422	59.0	3.23%	1087.2	2.29%	motif file (matrix)	svg

68	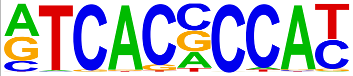	Srebp1a(bHLH)/HepG2-Srebp1a-ChIP-Seq(GSE31477)/Homer	1e-2	-4.972e+00	0.0448	50.0	2.73%	895.8	1.88%	motif file (matrix)	svg
69	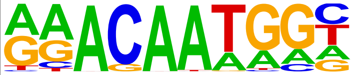	Sox15(HMG)/CPA-Sox15-ChIP-Seq(GSE62909)/Homer	1e-2	-4.960e+00	0.0448	184.0	10.06%	3995.2	8.40%	motif file (matrix)	svg
70	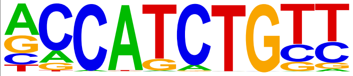	NeuroG2(bHLH)/Fibroblast-NeuroG2-ChIP-Seq(GSE75910)/Homer	1e-2	-4.956e+00	0.0448	370.0	20.23%	8544.5	17.97%	motif file (matrix)	svg
71	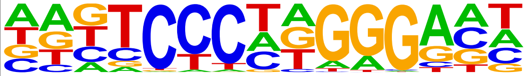	EBF2(EBF)/BrownAdipose-EBF2-ChIP-Seq(GSE97114)/Homer	1e-2	-4.952e+00	0.0448	230.0	12.58%	5105.8	10.74%	motif file (matrix)	svg
72	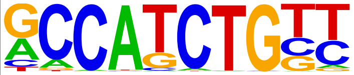	NeuroD1(bHLH)/Islet-NeuroD1-ChIP-Seq(GSE30298)/Homer	1e-2	-4.770e+00	0.0518	208.0	11.37%	4595.0	9.66%	motif file (matrix)	svg
73	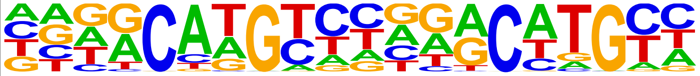	p63(p53)/Keratinocyte-p63-ChIP-Seq(GSE17611)/Homer	1e-2	-4.739e+00	0.0527	92.0	5.03%	1850.2	3.89%	motif file (matrix)	svg
74	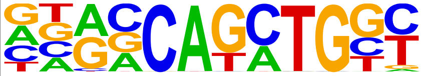	Atoh1(bHLH)/Cerebellum-Atoh1-ChIP-Seq(GSE22111)/Homer	1e-2	-4.707e+00	0.0537	276.0	15.09%	6259.7	13.16%	motif file (matrix)	svg
75	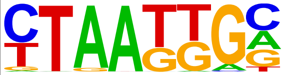	Isl1(Homeobox)/Neuron-Isl1-ChIP-Seq(GSE31456)/Homer	1e-2	-4.685e+00	0.0542	316.0	17.28%	7247.2	15.24%	motif file (matrix)	svg
76	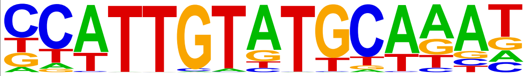	Oct4:Sox17(POU,Homeobox,HMG)/F9-Sox17-ChIP-Seq(GSE44553)/Homer	1e-2	-4.625e+00	0.0567	22.0	1.20%	327.2	0.69%	motif file (matrix)	svg

Supplemental Data 2: HOMER Motif Analysis from Extended Data Figure 6g. Homer Known Motif Enrichment Results (/media/tvlab/tb/2023_Elrod_H3K27ac/Figures/2023-02-13/2023-02-13_more_open_then_closed_99_regions)

Homer *de novo* Motif Results
Gene Ontology Enrichment Results
Known Motif Enrichment Results (txt file)
Total Target Sequences = 99, Total Background Sequences = 48694

Rank	Motif	Name	P-value	log P-pvalue	q-value (Benjamini)	# Target Sequences with Motif	% of Targets Sequences with Motif	# Background Sequences with Motif	% of Background Sequences with Motif	Motif File	SVG
1	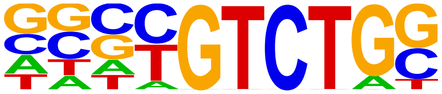	Smad4(MAD)/ESC-SMAD4-ChIP-Seq(GSE29422)/Homer	1e-4	-1.124e+01	0.0058	40.0	40.40%	10371.9	21.30%	motif file (matrix)	svg
2	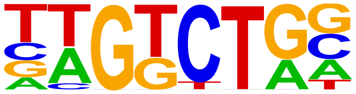	Smad3(MAD)/NPC-Smad3-ChIP-Seq(GSE36673)/Homer	1e-4	-9.474e+00	0.0169	53.0	53.54%	16786.5	34.47%	motif file (matrix)	svg
3	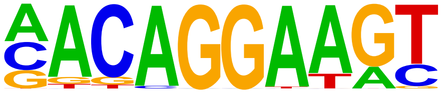	Ets1-distal(ETS)/CD4+-PolII-ChIP-Seq(Barski_et_al.)/Homer	1e-2	-6.386e+00	0.2472	9.0	9.09%	1340.8	2.75%	motif file (matrix)	svg
4	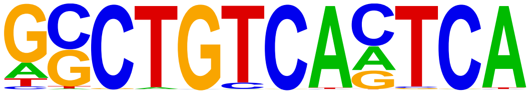	PBX1(Homeobox)/MCF7-PBX1-ChIP-Seq(GSE28007)/Homer	1e-2	-6.026e+00	0.2656	5.0	5.05%	453.4	0.93%	motif file (matrix)	svg
5	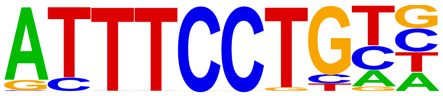	EWS:ERG-fusion(ETS)/CADO_ES1-EWS:ERG-ChIP-Seq(SRA014231)/Homer	1e-2	-5.744e+00	0.2817	12.0	12.12%	2373.8	4.87%	motif file (matrix)	svg
6	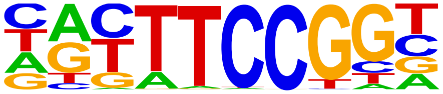	Elk4(ETS)/Hela-Elk4-ChIP-Seq(GSE31477)/Homer	1e-2	-5.382e+00	0.3373	16.0	16.16%	3839.0	7.88%	motif file (matrix)	svg
7	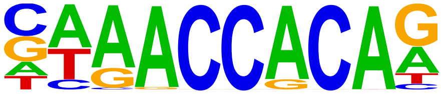	RUNX(Runt)/HPC7-Runx1-ChIP-Seq(GSE22178)/Homer	1e-2	-5.003e+00	0.4221	13.0	13.13%	2944.2	6.04%	motif file (matrix)	svg
8	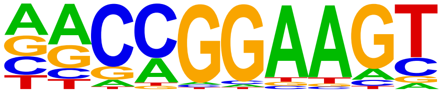	GABPA(ETS)/Jurkat-GABPa-ChIP-Seq(GSE17954)/Homer	1e-2	-4.686e+00	0.5072	19.0	19.19%	5262.0	10.80%	motif file (matrix)	svg
