# Supplementary material for: Nuclear ATP-citrate lyase regulates chromatin-dependent activation and maintenance of the myofibroblast gene program
Source: Nat Cardiovasc Res. 2024 Jul 5;3(7):869–82. doi: 10.1038/s44161-024-00502-3 (PMC11358007; doi:10.1038/s44161-024-00502-3)
Supplement: Supplementary file 2 — Reporting Summary [file 44161_2024_502_MOESM2_ESM.pdf]

nature portfolio

Corresponding author(s): John W. Elrod elrod@temple.edu

Last updated by author(s): May 27, 2024

Reporting Summary

Nature Portfolio wishes to improve the reproducibility of the work that we publish. This form provides structure for consistency and transparency in reporting. For further information on Nature Portfolio policies, see our Editorial Policies and the Editorial Policy Checklist.

Statistics

For all statistical analyses, confirm that the following items are present in the figure legend, table legend, main text, or Methods section.

n/a	Confirmed	
☐	☒	The exact sample size (*n*) for each experimental group/condition, given as a discrete number and unit of measurement
☐	☒	A statement on whether measurements were taken from distinct samples or whether the same sample was measured repeatedly
☐	☒	The statistical test(s) used AND whether they are one- or two-sided<br>*Only common tests should be described solely by name; describe more complex techniques in the Methods section.*
☐	☒	A description of all covariates tested
☐	☒	A description of any assumptions or corrections, such as tests of normality and adjustment for multiple comparisons
☐	☒	A full description of the statistical parameters including central tendency (e.g. means) or other basic estimates (e.g. regression coefficient) AND variation (e.g. standard deviation) or associated estimates of uncertainty (e.g. confidence intervals)
☐	☒	For null hypothesis testing, the test statistic (e.g. *F*, *t*, *r*) with confidence intervals, effect sizes, degrees of freedom and *P* value noted<br>*Give P values as exact values whenever suitable.*
☒	☐	For Bayesian analysis, information on the choice of priors and Markov chain Monte Carlo settings
☒	☐	For hierarchical and complex designs, identification of the appropriate level for tests and full reporting of outcomes
☒	☐	Estimates of effect sizes (e.g. Cohen's *d*, Pearson's *r*), indicating how they were calculated

Our web collection on statistics for biologists contains articles on many of the points above.

Software and code

Policy information about availability of computer code

Data collection	Illumina paired-end sequencing was performed by Novogene and provided raw FASTQ files. CUT&RUN data were processed using CUT-RUNTools-2.0 with references and details included in the Methods and Supplementary Table 4.
Data analysis	GraphPad Prism 10.2, Image Studio v5.2.5 (LICOR), CUT-RUNTools-2.0, DiffBind version 3.2.7, HOMER (v4.10), DAVID Functional Annotation Tool (v6.8)

For manuscripts utilizing custom algorithms or software that are central to the research but not yet described in published literature, software must be made available to editors and reviewers. We strongly encourage code deposition in a community repository (e.g. GitHub). See the Nature Portfolio guidelines for submitting code & software for further information.

Data

Policy information about availability of data

All manuscripts must include a data availability statement. This statement should provide the following information, where applicable:

- Accession codes, unique identifiers, or web links for publicly available datasets
- A description of any restrictions on data availability
- For clinical datasets or third party data, please ensure that the statement adheres to our policy

Source data and full length, uncropped western blots have been submitted to Nature and are available to readers. CUT&RUN sequencing data as fastq and bigWig files may be accessed from the NCBI GEO repository using accession number GSE232010.

Research involving human participants, their data, or biological material

Policy information about studies with human participants or human data. See also policy information about sex, gender (identity/presentation), and sexual orientation and race, ethnicity and racism.

Reporting on sex and gender	The human cardiac fibroblast samples using in Figure 6 were both from males.
Reporting on race, ethnicity, or other socially relevant groupings	The human cardiac fibroblast samples using in Figure 6 were both from Caucasian, middle aged subjects.
Population characteristics	Patients receiving heart transplants were presented the opportunity to donate their failing heart to research. Written informed consent was obtained from each heart transplant recipient.
Recruitment	Participants were recruited from the Hospital of University of Pennsylvania.
Ethics oversight	University of Pennsylvania Institutional Review Board.

Note that full information on the approval of the study protocol must also be provided in the manuscript.

Field-specific reporting

Please select the one below that is the best fit for your research. If you are not sure, read the appropriate sections before making your selection.

☒ Life sciences ☐ Behavioural & social sciences ☐ Ecological, evolutionary & environmental sciences

For a reference copy of the document with all sections, see nature.com/documents/nr-reporting-summary-flat.pdf

Life sciences study design

All studies must disclose on these points even when the disclosure is negative.

Sample size	In vitro experiments performed with a minimum of 3 biological replicates as is standard in biology. In vivo experiment sample size was determined using the nQuery Advisor 3.0 software (Statistical Solutions) for estimation of sample size and from previous experiments.
Data exclusions	A single data point in Fig, 3h was identified as an outlier by Grubbs test with an alpha of 0.05 and removed from subsequent data analysis. This identified outlier was included visually in graphs for data transparency (symbolized by an X). One sample from the CUT&RUN data that did not cluster close to its group (see Figure 5b) was removed from subsequent analysis.
Replication	For in vitro experiments, a minimum of 3 biological replicates each from separate isolations of fibroblasts were used. Where biological replicates are not appropriate for a given experiment, the experiment was replicated 3 times.
Randomization	Mice of respective genotypes were assigned randomly to sham or transverse aortic constriction groups. For in vitro experiments, samples were randomly assigned treatment groups.
Blinding	Researchers were blinded from the mice genotypes by a technician assigning numerical ear tags to each mouse. This method enabled surgeons and the experimenter to perform blinded in vivo experiments and cross-reference the numerical identifier with the genotype during data analysis. Images and echocardiographs were collected and analyzed from a blinded technician. For in vitro experiments, samples were number coded for blinding before being given to a technician to perform the terminal experiment.

Reporting for specific materials, systems and methods

We require information from authors about some types of materials, experimental systems and methods used in many studies. Here, indicate whether each material, system or method listed is relevant to your study. If you are not sure if a list item applies to your research, read the appropriate section before selecting a response.

Materials & experimental systems

n/a	Involved in the study	
☐	☒	Antibodies
☐	☒	Eukaryotic cell lines
☒	☐	Palaeontology and archaeology
☐	☒	Animals and other organisms
☒	☐	Clinical data
☒	☐	Dual use research of concern
☒	☐	Plants

Methods

n/a	Involved in the study	
☐	☒	ChIP-seq
☒	☐	Flow cytometry
☒	☐	MRI-based neuroimaging

Antibodies

Antibodies used	Histone H3K27ac Antibody, SNAP-ChIP® Certified, EpiCypher, 13-0045, rabbit, for CUT&RUN<br>CUTANA™ Rabbit IgG CUT&RUN Negative Control Antibody, EpiCypher, 13-0042, for CUT&RUN<br>Phospho ATP Citrate Lyase (Ser455) Antibody, Cell Signaling Technology, 4331S, rabbit for Western blot<br>ATP-Citrate Lyase, Abcam, Ab40793, rabbit, for western blot and co-IP<br>Alpha-Tubulin, Abcam, Ab7291, mouse, for western blot<br>Collagen type I alpha-1, Cell Signaling Technology, 72026S, rabbit, for western blot<br>Lamin B1, Abcam, Ab229025, rabbit, for western blot<br>Fibrillarin, Cell Signaling Technology, 2639S, rabbit, for western blot<br>Fatty Acid Synthase, Cell Signaling Technology, 3189S, rabbit, for western blot<br>GFP, Rockland, 600-101-215M, goat, for western blot<br>HDAC1, Cell Signaling Technology, 5356T, for western blot<br>POSTN, Abcam, ab14041, for western blot<br>LDHA, Cell Signaling Technology, 2012S, for western blot<br>Smad2/3, BD Biosciences, 610842, mouse, for western blot and co-IP<br>p300, Santa Cruz, sc-32244, mouse, for co-IP<br>anti-mouse IgG, Santa Cruz, sc-2025, mouse, for co-IP<br>H3K27ac, Abcam, ab4729, rabbit, for ChIP<br>Normal Rabbit IgG, PeproTech, 500-P00, rabbit, for ChIP
Validation	All antibodies were validated by the manufacturer and extensively referenced by third-party researchers. Literature provided by the manufacturer was examined to assess sufficient validation.

Eukaryotic cell lines

Policy information about cell lines and Sex and Gender in Research

Cell line source(s)	Primary murine cardiac fibroblasts isolated from male and femal C57BL/6NJ mice, which was used as a background strain for isolations of primary mouse embryonic fibroblasts and immortalized cardiac fibroblasts. The immortalized mouse cardiac fibroblast line was derived from a single male mouse with the Tcf21-MCM x Rosa26-TdTomato genotype.
Authentication	None of the cell types were authenticated.
Mycoplasma contamination	Cultured cells were not tested for mycoplasma infection.
Commonly misidentified lines (See ICLAC register)	No commonly misidentified cells lines were used.

Animals and other research organisms

Policy information about studies involving animals; ARRIVE guidelines recommended for reporting animal research, and Sex and Gender in Research

Laboratory animals	mus musculus, C57BL/6NJ mice, aged 12 weeks minimum to isolate cells from or enroll in in vivo study. Mice had cages, chow, and water checked regularly and changed as needed. 12 hour light-dark cycles. ambient temperature is roughly 78F (~26C) with about 60% humidity.
Wild animals	No wild animals used.
Reporting on sex	In vivo mouse studies used roughly equal numbers of male and female mice. All in vivo data analyzed male and female mice separately to examine the relevancy of sex. This study found no difference between male and female mice, so data of both sexes are aggregated.
Field-collected samples	No field collected samples.
Ethics oversight	Institutional Animal Care and Use Committee (IACUC)<br>Association for Assessment and Accreditation of Laboratory Animal Care International (AAALAC)<br>Temple University Laboratory Animal Resources (ULAR)

Note that full information on the approval of the study protocol must also be provided in the manuscript.

Plants

Seed stocks	Not applicable
Novel plant genotypes	Not applicable
Authentication	not applicable

ChIP-seq

Data deposition

☒ Confirm that both raw and final processed data have been deposited in a public database such as GEO.

☒ Confirm that you have deposited or provided access to graph files (e.g. BED files) for the called peaks.

Data access links
May remain private before publication.

NCBI GEO repository using accession number (GSE232010)

Files in database submission

ctrl_A1_CKDL230000952-1A_HT3LYDSX5_L3_1.fq.gz
ctrl_A1_CKDL230000952-1A_HT3LYDSX5_L3_2.fq.gz
ctrl_B2_CKDL230000953-1A_HT3LYDSX5_L3_1.fq.gz
ctrl_B2_CKDL230000953-1A_HT3LYDSX5_L3_2.fq.gz
ctrl_C3_CKDL230000954-1A_HT3LYDSX5_L3_1.fq.gz
ctrl_C3_CKDL230000954-1A_HT3LYDSX5_L3_2.fq.gz
ctrl_D4_CKDL230000955-1A_HT3LYDSX5_L3_1.fq.gz
ctrl_D4_CKDL230000955-1A_HT3LYDSX5_L3_2.fq.gz
ACLi_A6_CKDL230000957-1A_HT3LYDSX5_L3_1.fq.gz
ACLi_A6_CKDL230000957-1A_HT3LYDSX5_L3_2.fq.gz
ACLi_B7_CKDL230000958-1A_HT3LYDSX5_L3_1.fq.gz
ACLi_B7_CKDL230000958-1A_HT3LYDSX5_L3_2.fq.gz
ACLi_C8_CKDL230000959-1A_HT3LYDSX5_L3_1.fq.gz
ACLi_C8_CKDL230000959-1A_HT3LYDSX5_L3_2.fq.gz
ACLi_D9_CKDL230000960-1A_HT3LYDSX5_L3_1.fq.gz
ACLi_D9_CKDL230000960-1A_HT3LYDSX5_L3_2.fq.gz
TGFb_A11_CKDL230000962-1A_HT3LYDSX5_L3_1.fq.gz
TGFb_A11_CKDL230000962-1A_HT3LYDSX5_L3_2.fq.gz
TGFb_B12_CKDL230000963-1A_HT3LYDSX5_L3_1.fq.gz
TGFb_B12_CKDL230000963-1A_HT3LYDSX5_L3_2.fq.gz
TGFb_C13_CKDL230000964-1A_HT3LYDSX5_L3_1.fq.gz
TGFb_C13_CKDL230000964-1A_HT3LYDSX5_L3_2.fq.gz
TGFb_D14_CKDL230000965-1A_HT3LYDSX5_L3_1.fq.gz
TGFb_D14_CKDL230000965-1A_HT3LYDSX5_L3_2.fq.gz
ACLi_TGFb_B17_CKDL230000968-1A_HT3LYDSX5_L3_1.fq.gz
ACLi_TGFb_B17_CKDL230000968-1A_HT3LYDSX5_L3_2.fq.gz
ACLi_TGFb_C18_CKDL230000969-1A_HT3LYDSX5_L3_1.fq.gz
ACLi_TGFb_C18_CKDL230000969-1A_HT3LYDSX5_L3_2.fq.gz
ACLi_TGFb_D19_CKDL230000970-1A_HT3LYDSX5_L3_1.fq.gz
ACLi_TGFb_D19_CKDL230000970-1A_HT3LYDSX5_L3_2.fq.gz
ctrl_A1_CKDL230000952-1A_HT3LYDSX5_L3.cpm.norm.bw
ctrl_B2_CKDL230000953-1A_HT3LYDSX5_L3.cpm.norm.bw
ctrl_C3_CKDL230000954-1A_HT3LYDSX5_L3.cpm.norm.bw
ctrl_D4_CKDL230000955-1A_HT3LYDSX5_L3.cpm.norm.bw
ACLi_A6_CKDL230000957-1A_HT3LYDSX5_L3.cpm.norm.bw
ACLi_B7_CKDL230000958-1A_HT3LYDSX5_L3.cpm.norm.bw
ACLi_C8_CKDL230000959-1A_HT3LYDSX5_L3.cpm.norm.bw
ACLi_D9_CKDL230000960-1A_HT3LYDSX5_L3.cpm.norm.bw
TGFb_A11_CKDL230000962-1A_HT3LYDSX5_L3.cpm.norm.bw
TGFb_B12_CKDL230000963-1A_HT3LYDSX5_L3.cpm.norm.bw
TGFb_C13_CKDL230000964-1A_HT3LYDSX5_L3.cpm.norm.bw
TGFb_D14_CKDL230000965-1A_HT3LYDSX5_L3.cpm.norm.bw
ACLi_TGFb_B17_CKDL230000968-1A_HT3LYDSX5_L3.cpm.norm.bw
ACLi_TGFb_C18_CKDL230000969-1A_HT3LYDSX5_L3.cpm.norm.bw
ACLi_TGFb_D19_CKDL230000970-1A_HT3LYDSX5_L3.cpm.norm.bw
more_occupied_then_less_99_regions.bed

Genome browser session (e.g. UCSC)	No longer applicable.

Methodology

Replicates	Con, n=4; Con + ACLi, n=4; TGFb + Veh, n=4; TGFb + ACLY inhibitor, n=3
Sequencing depth	Paired end reads, minimum of 3 million read pairs per sample.
Antibodies	Histone H3K27ac Antibody, SNAP-ChIP® Certified, EpiCypher, 13-0045<br>CUTANA™ Rabbit IgG CUT&RUN Negative Control Antibody, EpiCypher, 13-0042
Peak calling parameters	CUT-RUNTools-2.0<br>read alignment = bowtie2, aligning to mouse genome mm10 at an 85-95% alignment rate<br>marking duplicate alignments = Picard's MarkDuplicates, removing duplicates with Samtools<br>peak calling = MACS2 callpeak function with -q 0.01 as a parameter to retain only peaks passing our statistical threshold of q<0.01.
Data quality	Differentially occupied peaks were classified as those passing FDR<0.05.
Software	CUT-RUNTools-2.0, DiffBind version 3.2.7, HOMER version 4.10
