# Supplementary material for: Nuclear ATP-citrate lyase regulates chromatin-dependent activation and maintenance of the myofibroblast gene program
Source: Nat Cardiovasc Res. 2024 Jul 5;3(7):869–82. doi: 10.1038/s44161-024-00502-3 (PMC11358007; doi:10.1038/s44161-024-00502-3)

Full Blots For Figure 1

- 1a

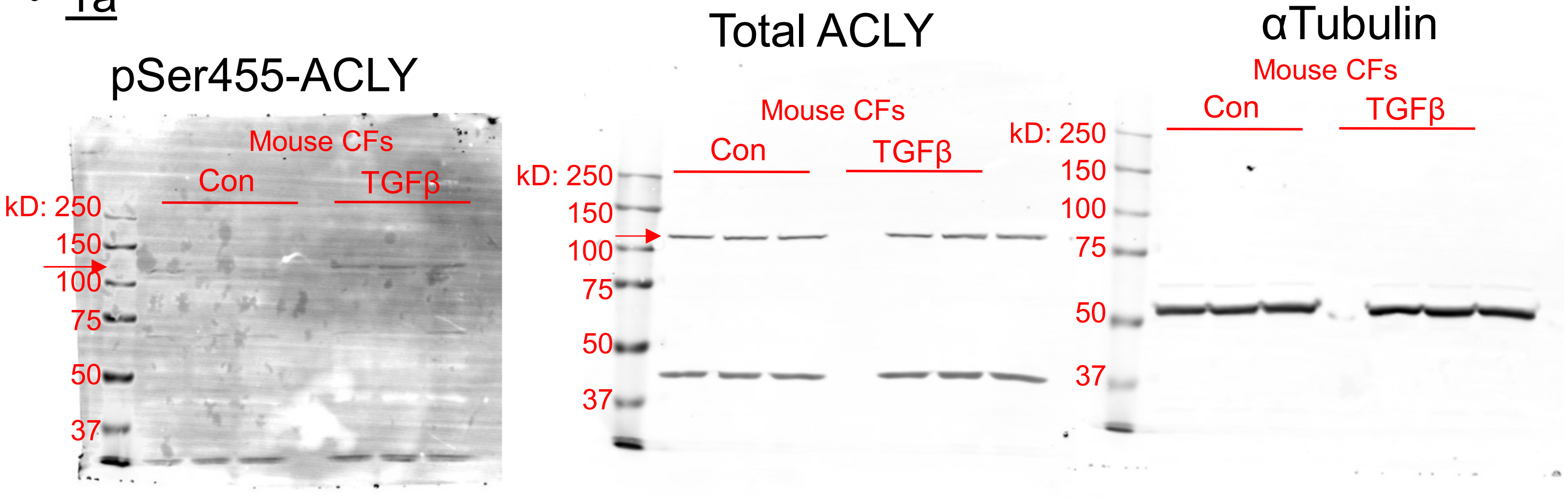

- 1e

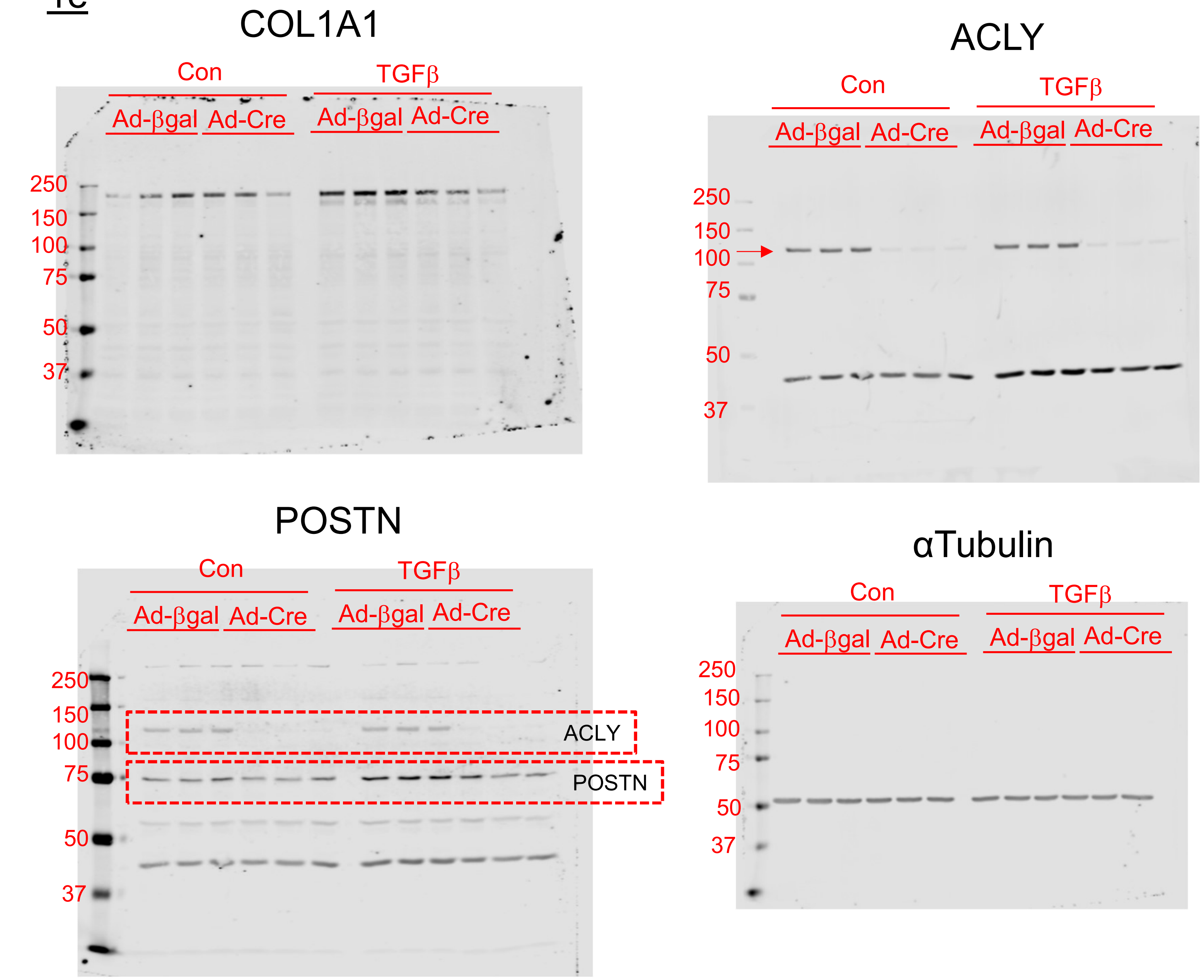

Full Blots For Figure 1c

Rep 1

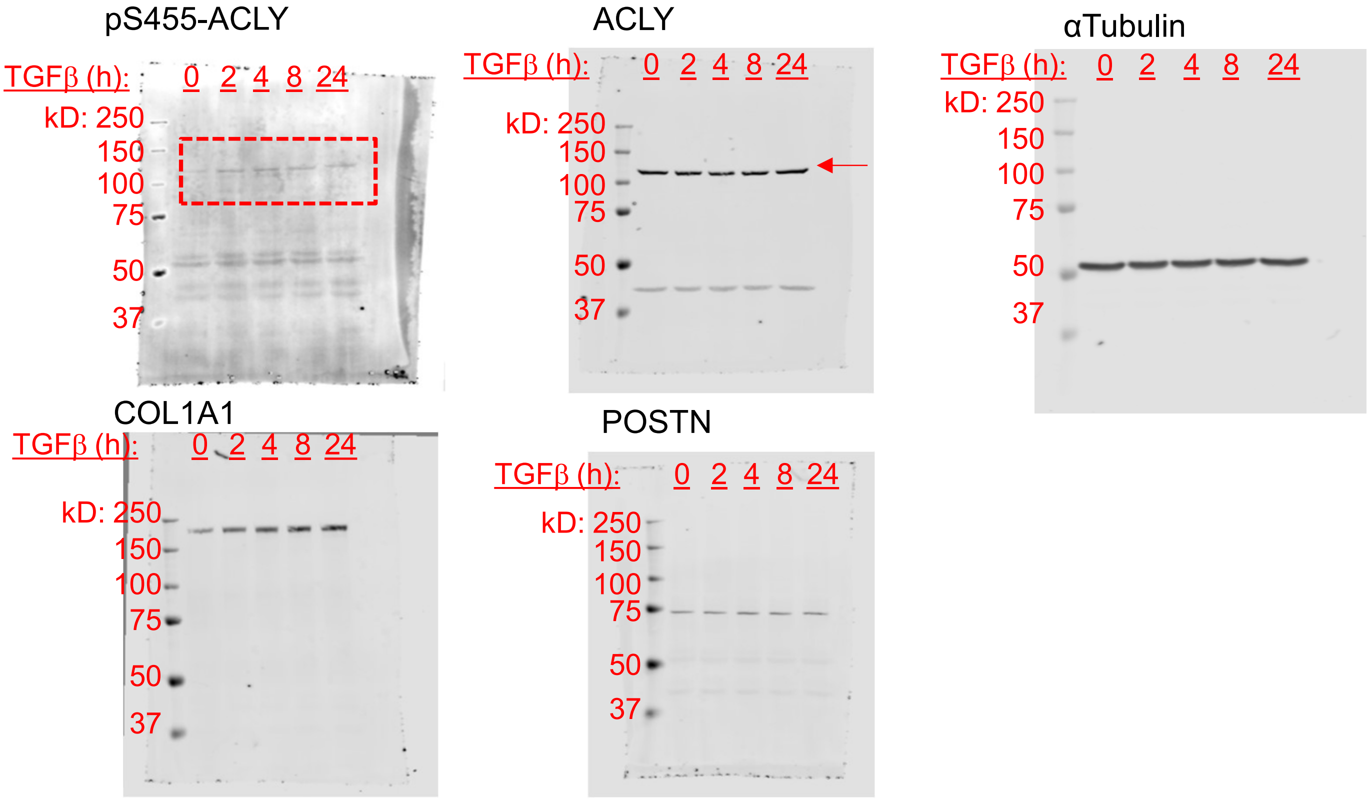

Full Blots For Figure 1c

Rep 2

pS455-ACLY

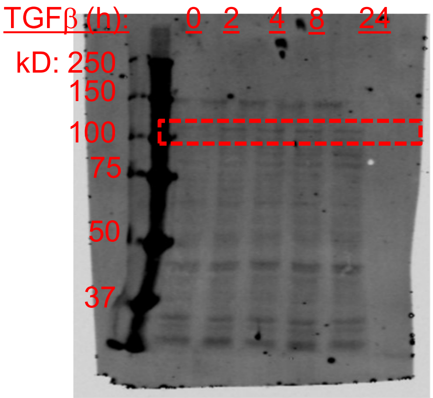

ACLY

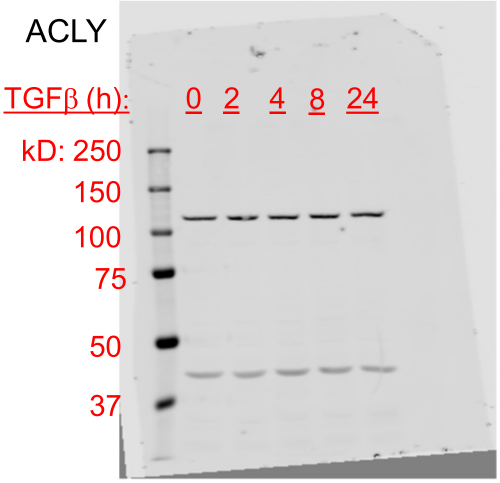

αTubulin

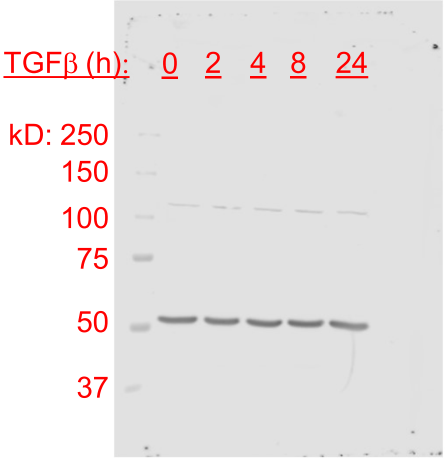

COL1A1

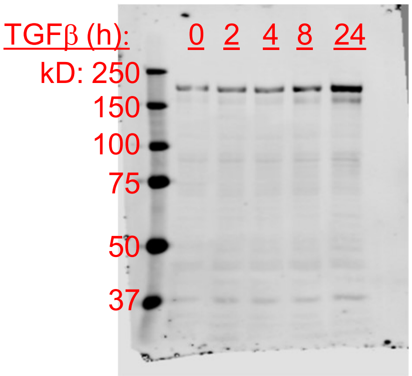

POSTN

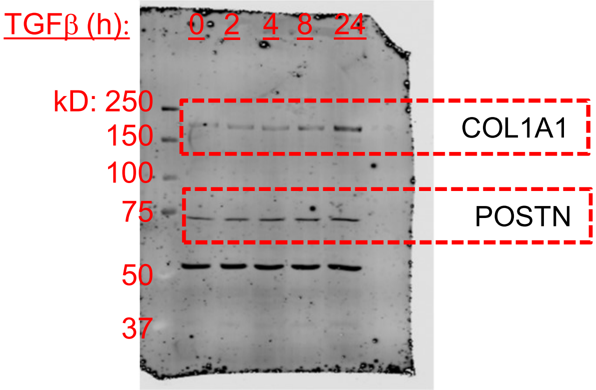

Rep 3

pS455-ACLY

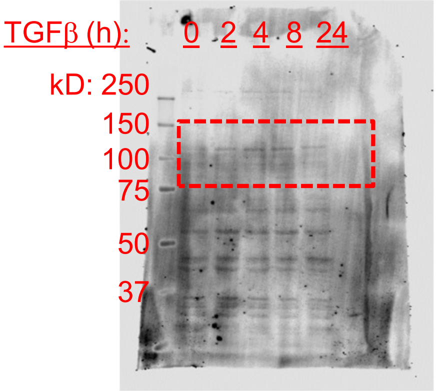

ACLY

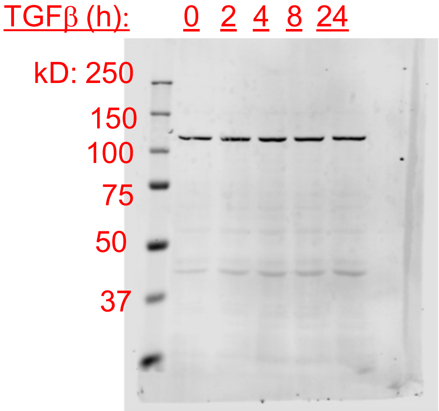

αTubulin

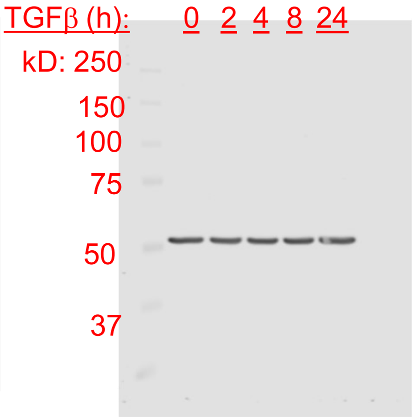

COL1A1

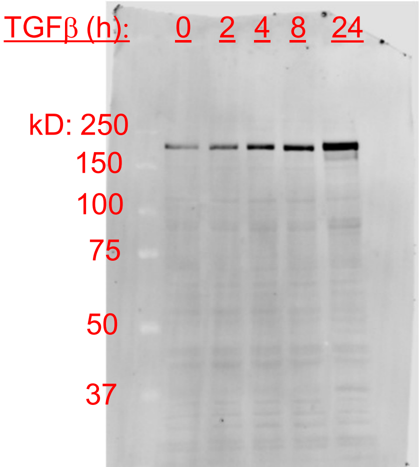

POSTN

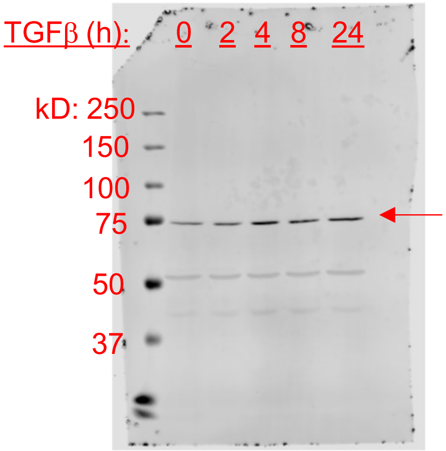

Full Blots For Figure 1

- 1j

COL1A1

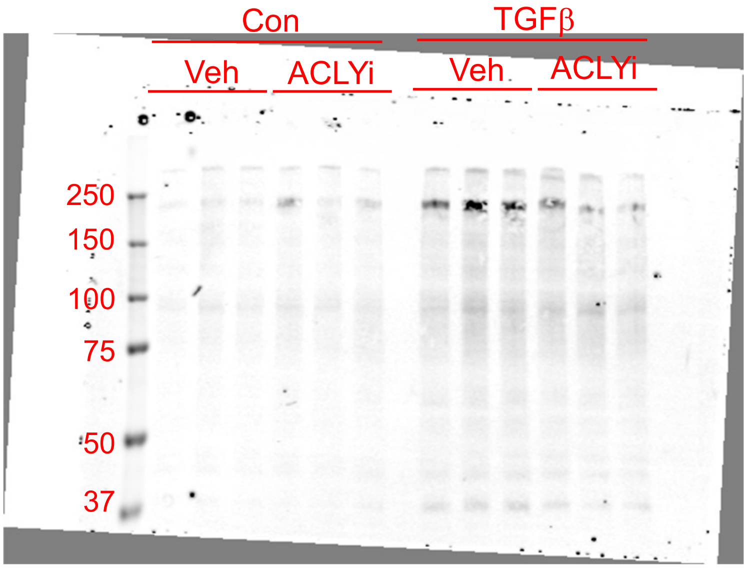

αTubulin

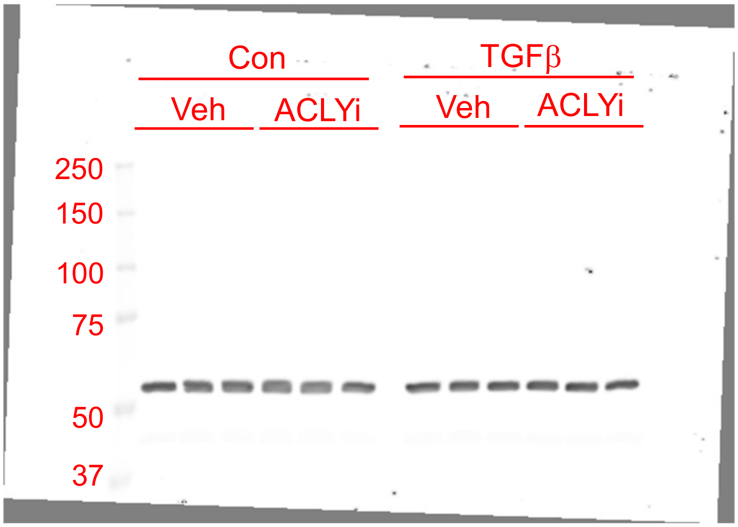

Supplement: Supplementary file 4 — Full-length western blots. [file 44161_2024_502_MOESM4_ESM.pdf]
