# Supplementary figures and images for: Nuclear ATP-citrate lyase regulates chromatin-dependent activation and maintenance of the myofibroblast gene program
Source: Nat Cardiovasc Res. 2024 Jul 5;3(7):869–82. doi: 10.1038/s44161-024-00502-3 (PMC11358007; doi:10.1038/s44161-024-00502-3)

• 2e

Col1a1

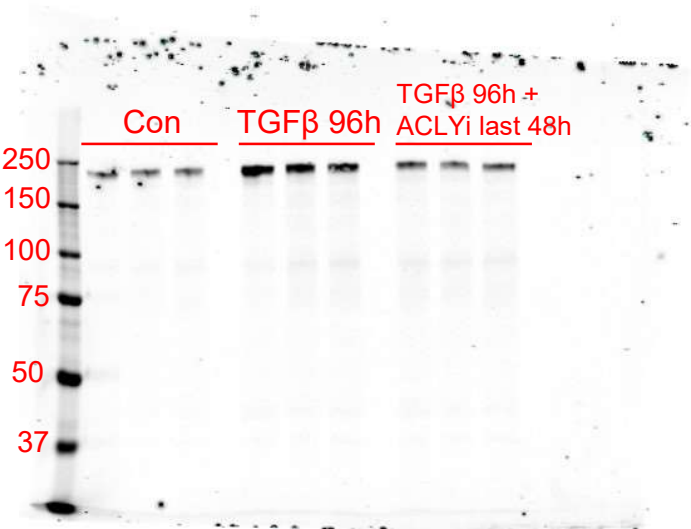

αTubulin

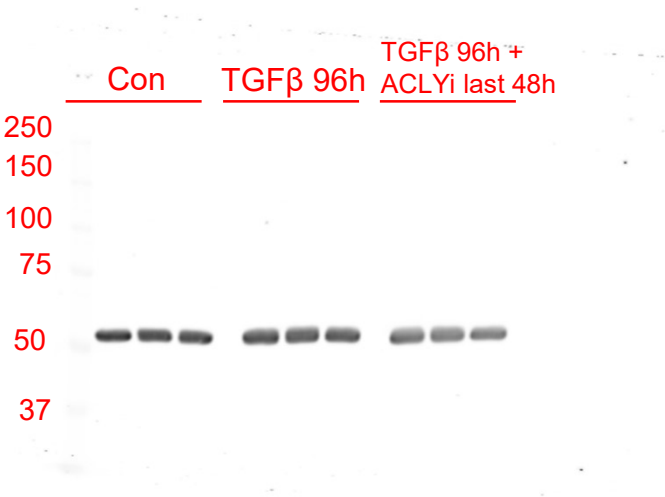

Supplement: Supplementary file 5 — Full-length western blots. [file 44161_2024_502_MOESM5_ESM.pdf]

Full Blots For Figure 3

• 3c

ACLY

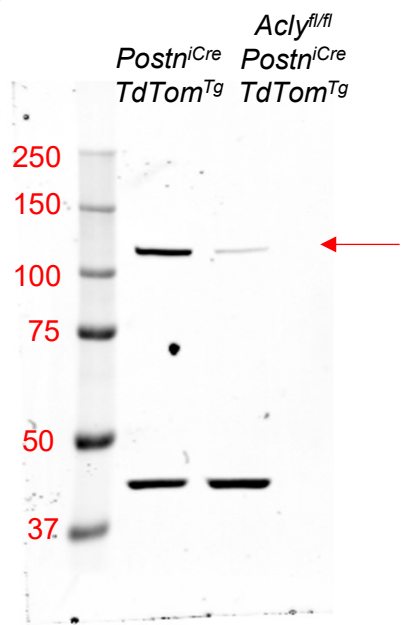

$\alpha$ Tubulin

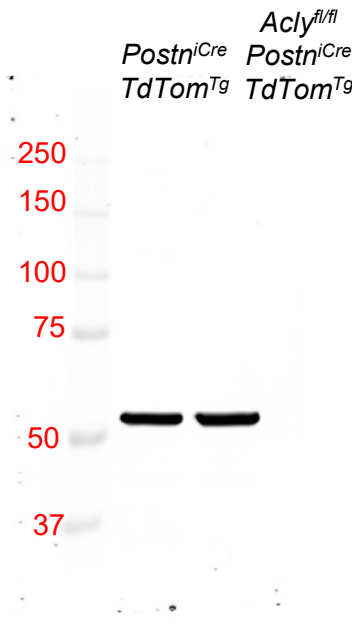

Supplement: Supplementary file 6 — Full-length western blots. [file 44161_2024_502_MOESM6_ESM.pdf]

Full Blots For Figure 4

• 4a

Rep 1

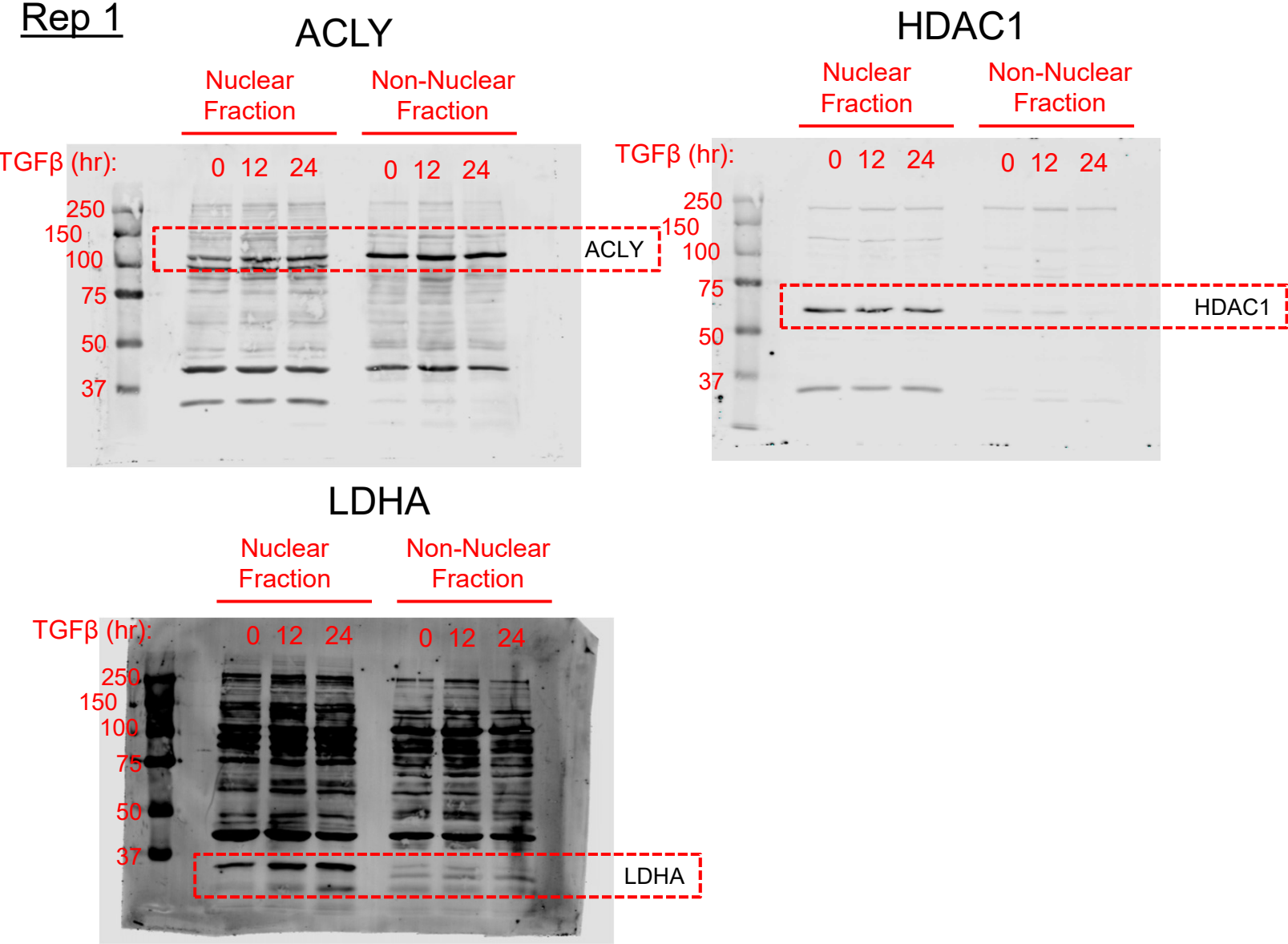

Full Blots For Figure 4

• 4a

Rep 2

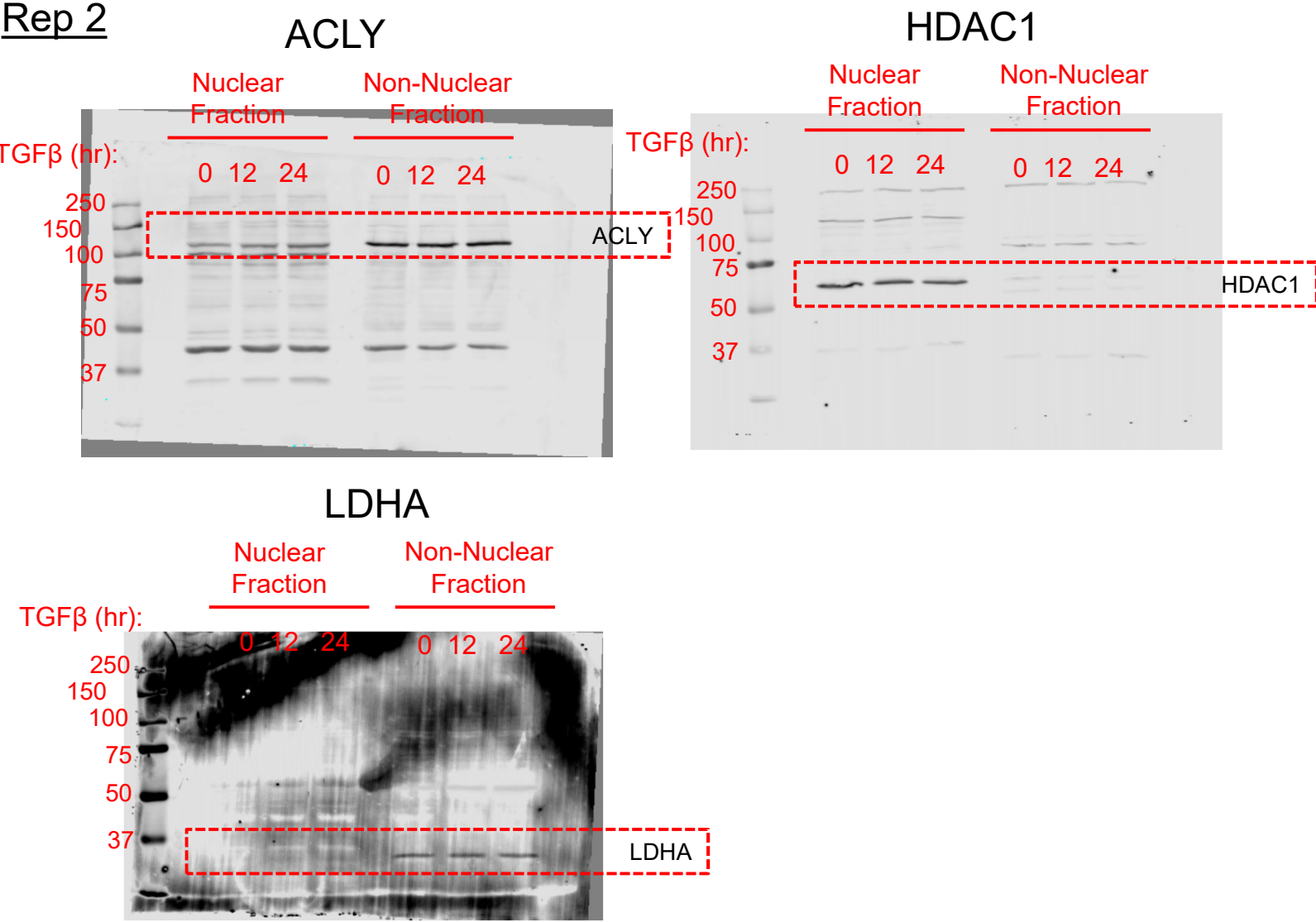

Full Blots For Figure 4

• 4a

Rep 3

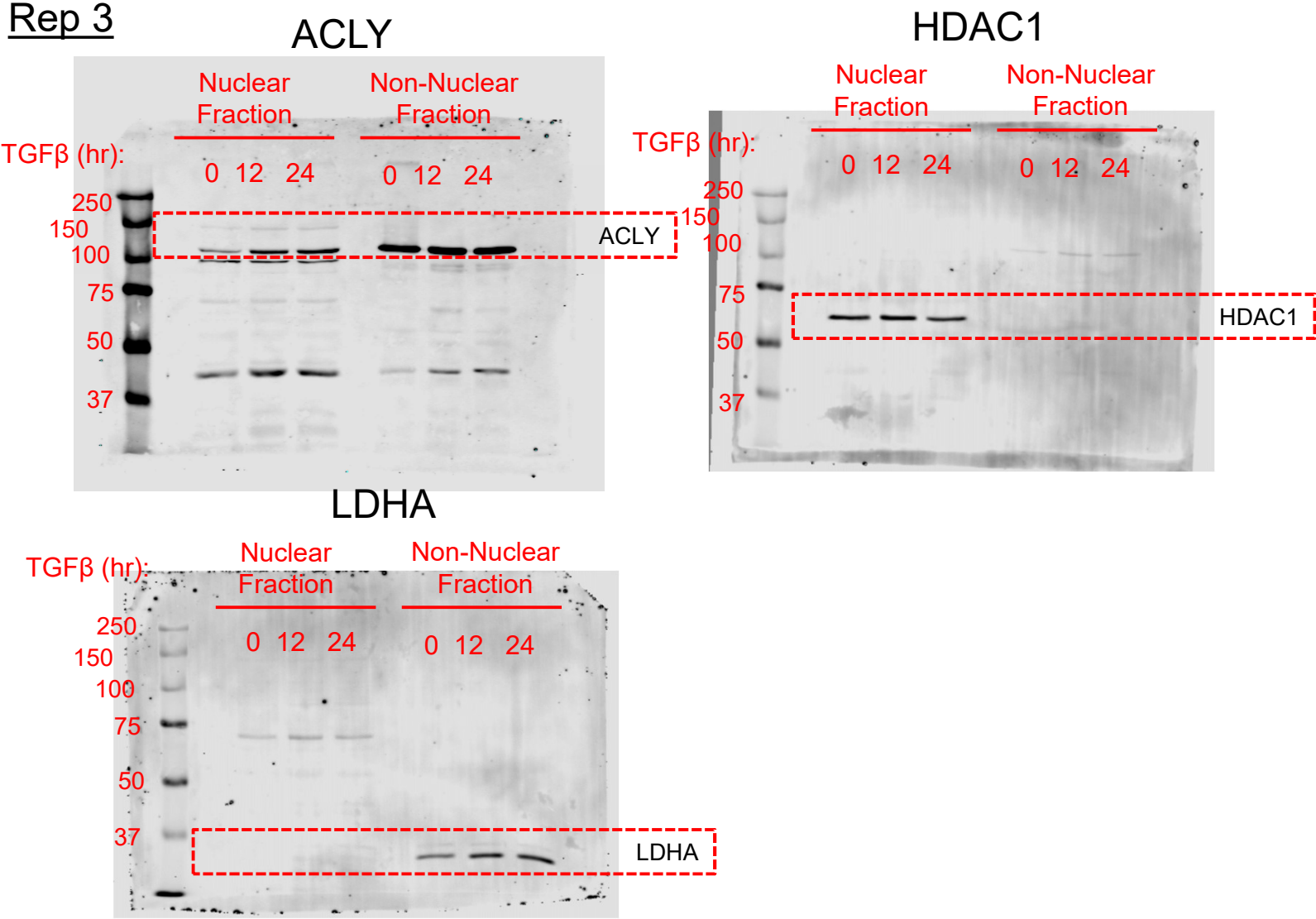

Supplement: Supplementary file 7 — Full-length western blots. [file 44161_2024_502_MOESM7_ESM.pdf]

- 5j

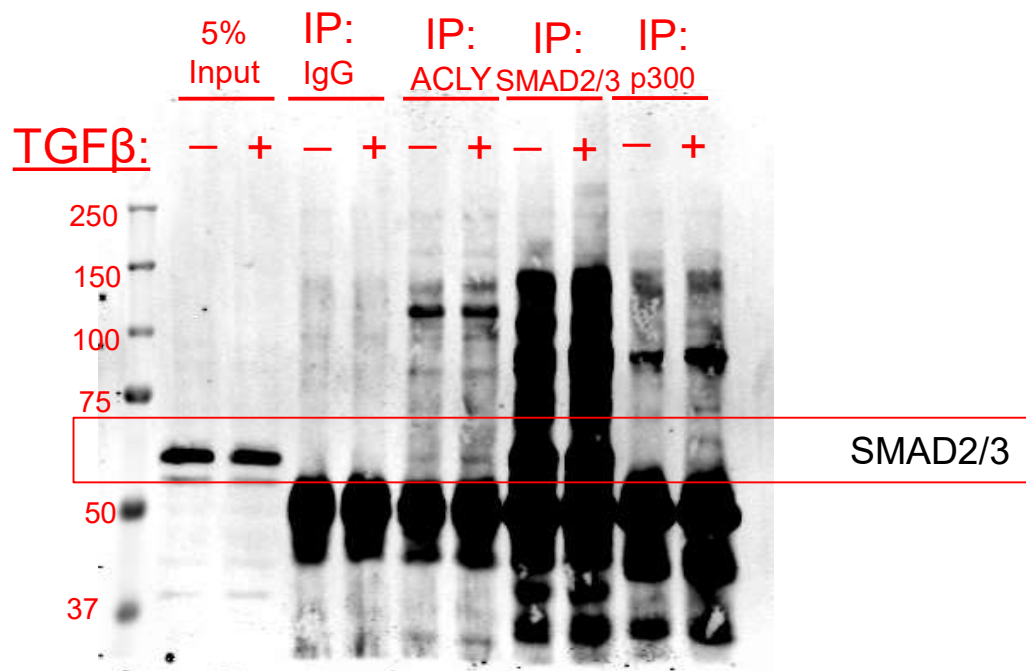

Supplement: Supplementary file 8 — Full-length western blots. [file 44161_2024_502_MOESM8_ESM.pdf]
