# Supplementary material for: Nuclear ATP-citrate lyase regulates chromatin-dependent activation and maintenance of the myofibroblast gene program
Source: Nat Cardiovasc Res. 2024 Jul 5;3(7):869–82. doi: 10.1038/s44161-024-00502-3 (PMC11358007; doi:10.1038/s44161-024-00502-3)

Full Blots For
Extended Figure 3a

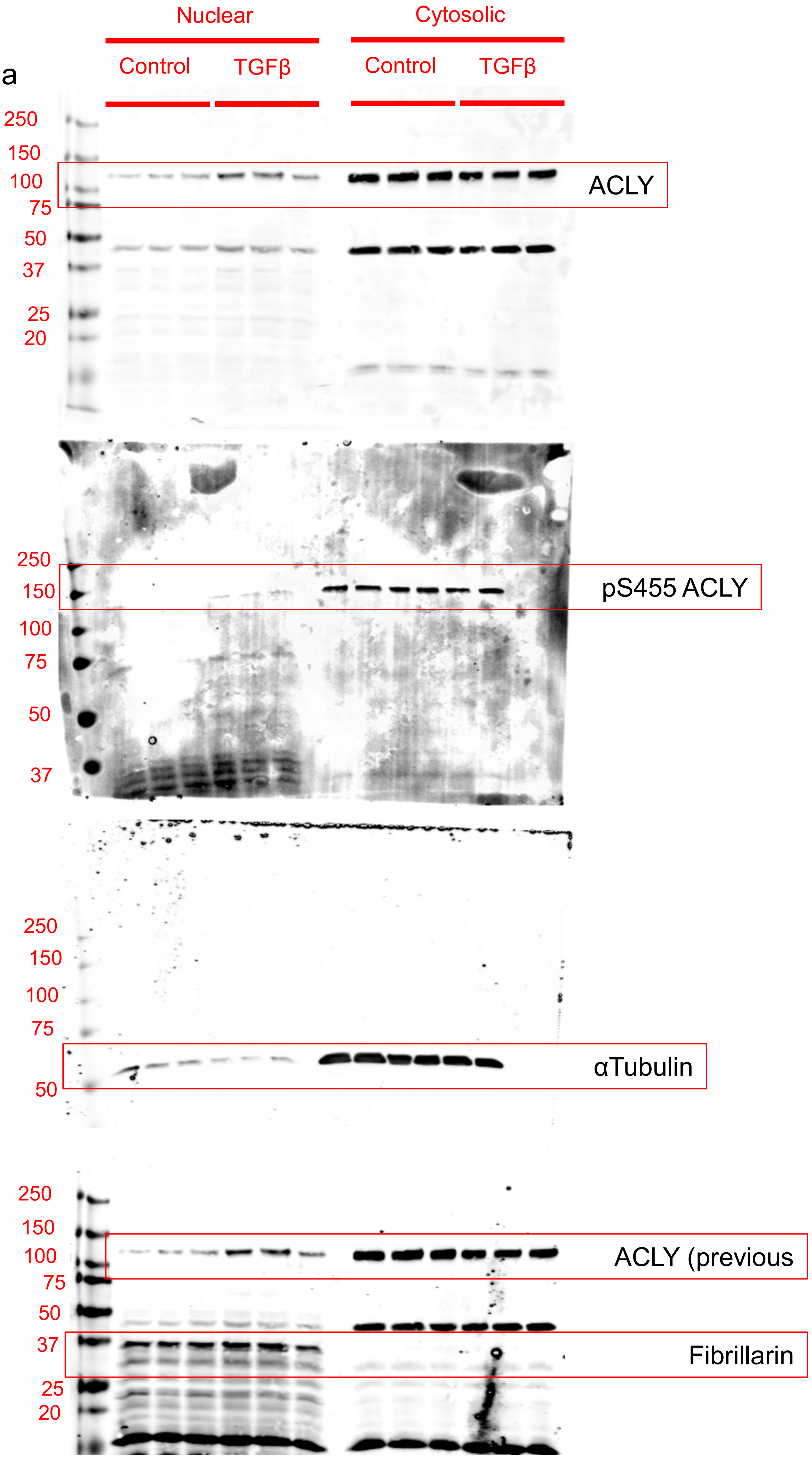

Full Blots For Extended Figure 3b

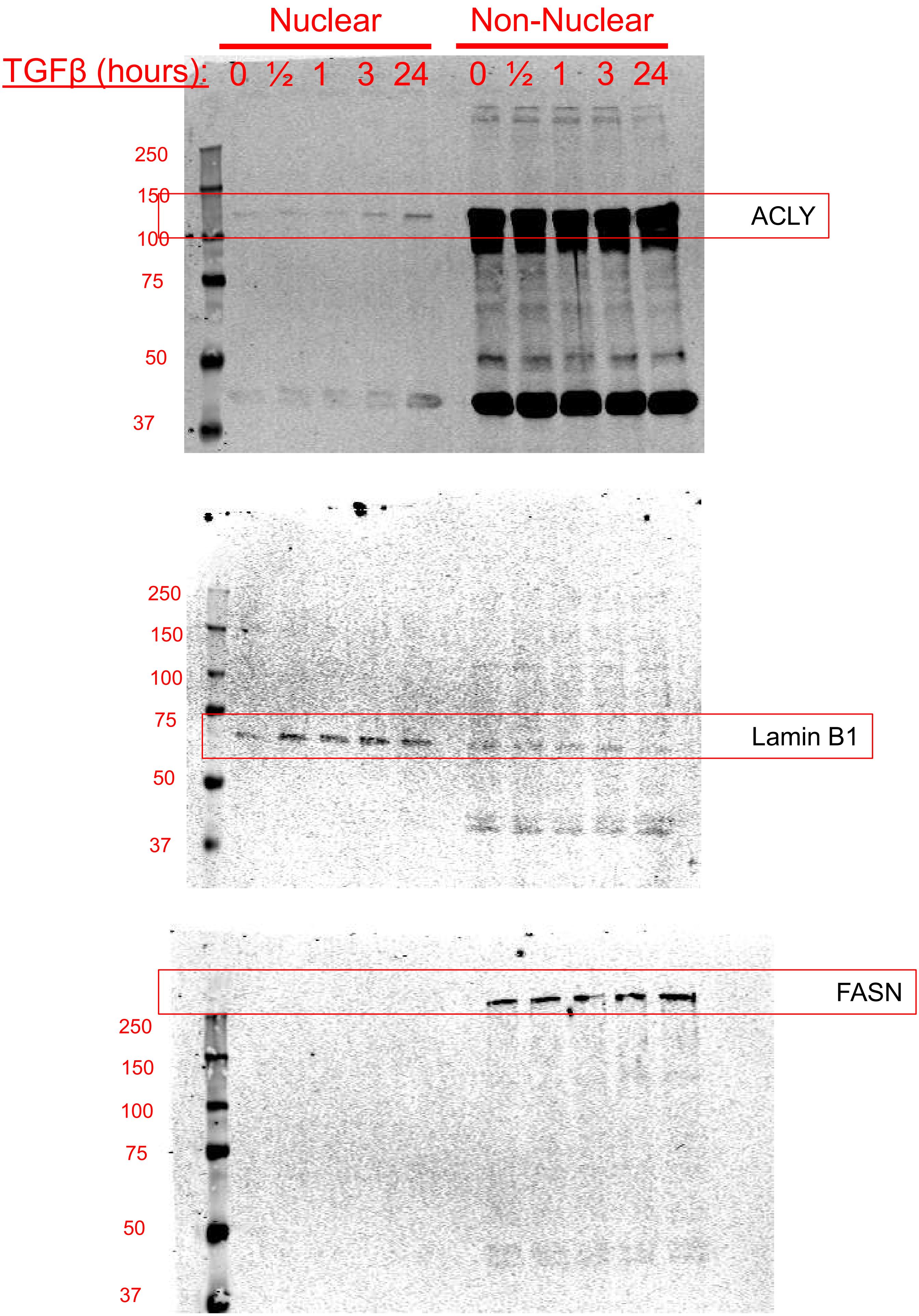

Full Blots For Extended Figure 3e

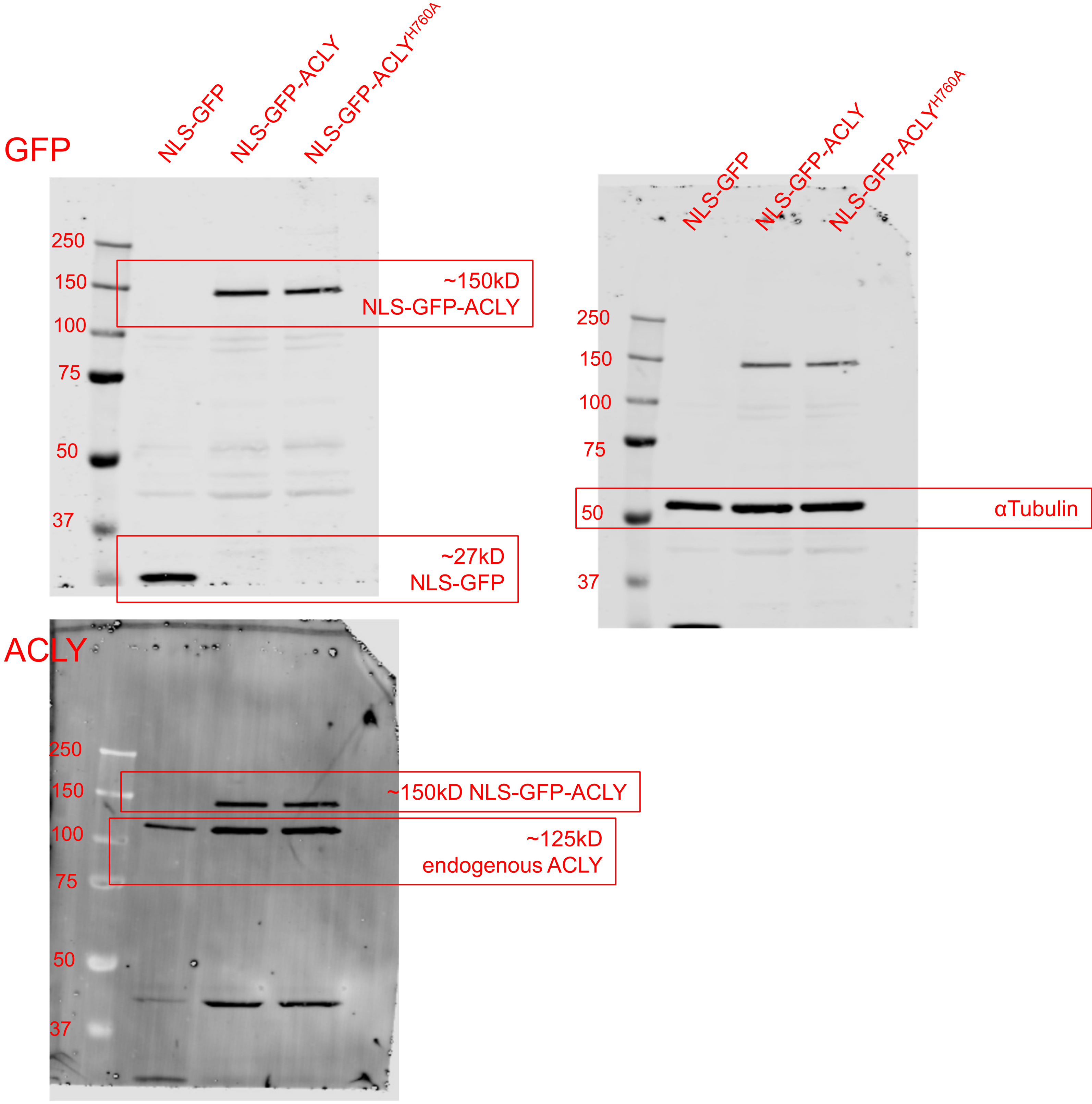

Supplement: Supplementary file 9 — Full-length western blots. [file 44161_2024_502_MOESM9_ESM.pdf]
